# Sex-Dependent Determinants of Uremic Toxicity in Chronic Kidney Disease

**DOI:** 10.3390/toxins18060242

**Published:** 2026-05-25

**Authors:** Oriana Nobus, Aurélie Carlier, Silvia M. Mihăilă, Vanessa Dubois

**Affiliations:** 1 Basic and Translational Endocrinology (BaTE) Laboratory, Department of Basic and Applied Medical Sciences, Faculty of Medicine and Health Sciences, Ghent University, 9000 Ghent, Belgium; oriana.nobus@ugent.be; 2 Maastricht Centre for Systems Biology and Bioinformatics (MaCSBio), Maastricht University, 6200 Maastricht, The Netherlands; a.carlier@maastrichtuniversity.nl; 3 Division of Pharmacology, Utrecht Institute for Pharmaceutical Sciences, Utrecht University, 3584 Utrecht, The Netherlands; s.mihaila@uu.nl

**Keywords:** chronic kidney disease, uremic toxins, uremic toxicity, sex hormones, sexual dimorphism

## Abstract

Chronic kidney disease (CKD) is characterized by the progressive accumulation of uremic toxins (UTs), which contribute to systemic complications, increased cardiovascular risk, and disease progression. Epidemiological and experimental evidence demonstrate pronounced sex differences in CKD progression and outcomes, yet the mechanisms underlying sex-specific uremic toxicity remain unclear. This review synthesizes current knowledge on sex differences in the origin, metabolism, transport, and biological effects of UTs, with a focus on sex-dependent regulatory mechanisms along the gut–liver–kidney axis. Sex hormones influence key determinants of toxin handling, including gut microbiota composition, hepatic enzyme activity, plasma protein binding, membrane transporter expression, and intracellular signaling pathways. Together, these factors regulate systemic toxin exposure and tissue susceptibility to injury. CKD also disrupts endocrine homeostasis, creating bidirectional interactions between hormonal regulation and toxin accumulation. Experimental and limited clinical evidence suggest that sex may influence circulating toxin profiles and susceptibility to toxin-associated complications. Collectively, sex is an important modulator of uremic toxicity, with sex hormones mediating at least part of the sex differences. A sex-informed framework may improve fundamental understanding through mechanistic studies and future clinical research may help clarify its relevance for biomarker development and support the development of personalized therapeutic strategies for CKD.

## 1. Introduction

Chronic kidney disease (CKD) is a major global health problem affecting approximately 10% of the adult population worldwide. With the ongoing aging population, CKD is increasingly considered as a silent epidemic [1,2]. CKD is defined by structural or functional kidney abnormalities, with or without reduced estimated glomerular filtration rate (eGFR), or by a GFR < 60 mL/min/1.73 m^2^ persisting for at least three months, irrespective of kidney damage [3,4,5]. Because symptoms often remain absent until advanced stages, CKD is frequently underdiagnosed [6,7]. Progressive loss of kidney function impairs the clearance of endogenous metabolites and exogenous compounds, leading to their accumulation in the circulation and the development of uremic syndrome [8]. This syndrome reflects a complex multisystem disorder resulting from the retention of biologically active solutes, collectively termed uremic solutes [9]. Their retention results mainly from reduced kidney glomerular filtration and clearance and is, in some cases, accompanied by a rise in generation in the kidney [10]. Many of these uremic solutes exert biological activity (toxicity) that adversely affects cellular and organ function and are then referred to as uremic toxins (UTs). In particular, plasma levels of protein-bound uremic toxins (PBUTs) increase with CKD progression [11,12].

UT accumulation plays a central role in CKD pathophysiology and associated complications [13]. Many UTs originate from endogenous metabolism and gut microbial activity, and contribute to inflammation, oxidative stress, endothelial dysfunction, and fibrosis [14]. Consequently, elevated circulating UT levels have been strongly associated with a higher risk of cardiovascular (CV) events, which is the leading cause of mortality in CKD patients [15,16,17]. Beyond CV complications, UTs are implicated in the development of neurological disorders (e.g., cognitive impairment and anxiety) and accelerate CKD progression itself [18,19,20,21]. UTs could also play a role in the crosstalk between bone and vessel function, and potentially contribute to the development of vascular and bone remodeling disorders in CKD patients [22]. Moreover, evidence indicates that UTs contribute to a range of symptoms associated with uremic syndrome, referred to as the uremic symptom burden [3,17,18,19,22,23,24,25]. Although hemodialysis reduces CKD-related mortality by removing some UTs, conventional dialysis poorly eliminates PBUTs, and current kidney replacement therapies fail to fully restore physiological toxin clearance [18,26,27]. Consequently, UTs remain key contributors to CKD-related morbidity and mortality and may also serve as biomarkers of early tubular kidney dysfunction [28,29,30].

Substantial sex differences exist in kidney physiology and CKD progression [31,32]. Although CKD prevalence is higher in women, men generally show faster disease progression, earlier onset of end stage kidney disease (ESKD), and higher mortality [33,34,35,36,37]. These differences have been linked to variations in kidney hemodynamics, kidney mass, transporter expression and hormonal regulation, particularly the effects of estrogens and androgens [36,38,39,40]. Importantly, these sex differences are not solely attributed to biological sex, i.e., traits typically assigned male or female at birth, and to the associated hormonal profiles, but are also influenced by gender-related socio-cultural factors such as healthcare access, treatment strategies, and disease recognition [35,41]. The CKD symptom burden, including nausea, fatigue, daytime sleepiness, impaired concentration, and pain, is known to increase with age and has been reported to be substantially higher in women than in men, with a more pronounced negative impact on the quality of life [24,42]. These findings suggest that sex-related differences in CKD extend beyond disease prevalence and progression and may also involve differences in symptom burden and underlying biological drivers.

Despite these observations, the modulating role of sex in UT generation, metabolism, clearance, and biological effects, i.e., uremic toxicity, has received limited attention. Multiple biological factors showing sex-specific variation, such as sex hormones, body composition, gut microbiota composition, inflammatory responses, and membrane transporter expression, regulate key processes involved in UT handling and biological activity [1,8,43,44,45]. These processes are integrated within the gut–liver–kidney axis, which governs toxin production, metabolism, distribution, and elimination [46,47,48,49]. Sex differences at any of these levels may contribute to the observed sexual dimorphism in CKD progression and complications [50,51,52].

A better understanding of sex-related determinants of uremic toxicity can help to clarify mechanisms underlying the sexual dimorphism in CKD and identify opportunities for more personalized, sex-informed approaches in diagnosis, symptom management, and therapeutic intervention. Of note, in this review the term sexual dimorphism refers specifically to differences between the two primary biological sexes, i.e., male (XY) and female (XX). However, we acknowledge that biological sex exists beyond a strict binary dichotomy and encompasses variations such as Turner syndrome (X), Klinefelter syndrome (XXY), and XYY or XXXY syndromes. While these chromosomal variations likely influence metabolic and kidney profiles, data regarding their specific impact on uremic toxicity are currently lacking. Here, we therefore focused on the potential mechanisms underlying the differences between the two primary biological sexes. In particular, sex hormones such as estrogens and androgens play a central regulatory role in modulating physiological processes relevant to uremic toxicity. These hormones influence gut microbial composition, hepatic metabolic enzyme activity, plasma protein binding, transporter expression, and cellular signaling pathways, all of which govern UT handling and toxicity [36,53,54,55,56,57,58,59,60]. Furthermore, CKD itself disrupts endocrine homeostasis, creating a bidirectional interaction between kidney dysfunction and hormonal regulation. Understanding these complex interactions is essential for elucidating sex-specific mechanisms underlying uremic toxicity.

Although sex differences in CKD and individual aspects of UT biology have been studied separately, an integrated mechanistic framework linking sex-dependent regulation to uremic toxicity is lacking. This review therefore provides a comprehensive overview of sex-dependent determinants of uremic toxicity and their relevance for CKD pathophysiology and patient care. We discuss sexual dimorphism in CKD, summarize current concepts regarding UT classification and metabolism, and examine how sex hormones may influence UT generation, metabolism, clearance, and toxicity across the gut–liver–kidney axis. While multiple mechanisms (e.g., sex hormones, lifestyle, sex chromosomes, …) most likely contribute to the observed sex differences, within this review we focus on the role of sex hormones as modulators of uremic toxicity. Finally, we address the potential clinical implications of sex-related differences in uremic toxicity for risk stratification and therapeutic management in CKD. Overall, this review uniquely integrates sex-specific regulation of UT biology across the gut–liver–kidney axis, with a particular focus on the distinct mechanistic levels at which sex differences may influence uremic toxicity in CKD.

## 2. Sex Differences in CKD-Associated Uremic Toxicity

CKD exhibits pronounced sex differences in incidence, progression, and clinical outcomes [35,36,37,61]. These differences likely arise from a combination of lifestyle, genetics, epigenetics, sex chromosomes, sex hormones, and sociocultural gender-related factors [52,60]. Among these, sex hormones are particularly relevant because they directly regulate pathways involved in UT generation, clearance, and toxicity. Understanding their effects on kidney physiology is therefore essential to interpret sex differences in uremic toxicity. In the following sections, we discuss how sex hormones influence CKD pathophysiology and UT handling, emphasizing the bidirectional interaction between endocrine regulation and kidney dysfunction.

### 2.1. Sex Hormone Biology and Their Effects on Kidney Structure and Function

Sex hormones, including estrogens, androgens, and progestogens, exert their effects through membrane-bound and intracellular receptors that activate transcription-dependent and independent signaling pathways [36]. This review focuses on estrogens and androgens. Although estrogens and androgens were traditionally associated with female and male physiology, respectively, both hormone classes regulate important biological and pathological processes in both sexes [36,62,63,64].

Sex hormones circulate either freely or bound to sex hormone-binding globulin (SHBG) or albumin and act locally and systemically on target tissues [65]. Estrogen receptors (ERα and ERβ) and androgen receptors (ARs) are widely expressed in the mammalian kidney, indicating an important role for sex hormones in kidney physiology and pathology [66,67,68,69,70]. Animal studies suggest that estrogens support preservation of normal kidney function, whereas excess testosterone accelerates kidney injury and fibrosis, highlighting the importance of hormonal balance for kidney health [71,72]. In addition, the male kidney serves as a site of testosterone catabolism, while the female kidney contributes to testosterone production, consistent with the observation that the human female kidney functions as an androgen-producing organ [73]. Consequently, as was shown in rats models, female kidneys depend on high circulating estrogen levels together with relatively low androgen concentrations to maintain homeostasis of steroid hormones [74].

Sexual dimorphism is also evident in kidney structure. Male mammals generally exhibit larger kidneys than females [51,75]. Furthermore, female mice not only have smaller kidneys, but also shorter proximal tubules than males [76]. These structural differences are largely attributed to androgen-driven hypertrophy of proximal tubular cells (PTC) rather than differences in glomerular number, suggesting that hormonal regulation primarily affects tubular structure and metabolic capacity. A recent study in mice showed that estrogens may additionally protect podocyte integrity and support glomerular resilience, potentially contributing to slower CKD progression in females [77]. However, it is important to note that most of these data come from animal models, particularly rodents, while only a limited amount of research is available in humans [66]. Because PTCs play a central role in transporter-mediated UT secretion, sex-related structural differences may influence toxin clearance and intracellular toxin exposure, thereby contributing to sex differences in UT handling and toxicity in CKD.

Animal studies further demonstrate sex differences in transporter expression, tubular handling, oxidative stress, nitric oxide (NO) metabolism, and regulation of the renin–angiotensin–aldosterone system (RAAS) [41,76,78,79,80,81,82,83,84]. Males generally show greater RAAS activation and stronger hypertensive responses to angiotensin II, mechanisms closely linked to fibrosis, CKD progression, and CV complications [84]. These findings align with epidemiological observations showing faster CKD progression and higher mortality in men despite a higher CKD prevalence in women. Together, these data support the concept that sex is an important determinant of uremic toxicity in CKD, although much of the current evidence remains preclinical.

### 2.2. Differential Effects of Sex Hormones on Kidney Health

Observed sex differences in CKD progression and outcomes suggest that sex hormones may influence kidney health and disease susceptibility. Understanding the differential effects of estrogens and androgens on kidney health may therefore provide mechanistic insight into these clinical patterns [60,85].

Estradiol, the most active estrogen, is generally considered protective, whereas testosterone has often been associated with kidney injury and fibrosis [36,86,87,88]. These protective effects are mediated through estrogen-dependent modulation of oxidative stress, inflammatory signaling, mitochondrial function, and fibrotic signaling, and RAAS activity, all of which are central mechanisms in uremic toxicity and CKD progression [52]. While estradiol exerts inhibitory effects on RAAS activity in mice and as such has a protective effect on hypertension [89], testosterone positively modulates the RAAS in mice, potentially predisposing them to hypertension and kidney dysfunction [56]. In addition, testosterone may directly contribute to local inflammatory processes within the kidney [56]. Nevertheless, recent clinical evidence indicates that testosterone may also protect kidney function when endogenous levels are relatively low [36,90,91], suggesting concentration-dependent and context-specific effects of androgens in CKD [52].

Interpretation of androgen effects is further complicated by aromatization of testosterone into estradiol [60,92]. This conversion may partly explain why lower testosterone levels are associated with reduced kidney function (i.e., lower eGFR) in men but with improved kidney function in women (i.e., higher eGFR) [93,94]. In men, declining testosterone may therefore also reflect reduced estradiol availability. Hormonal balance between estrogens and androgens appears particularly in postmenopausal women, in whom hormonal fluctuations may accelerate kidney disease progression [57,95]. Furthermore, studies using animal models of natural aging support the idea that estrogens slow progression of CKD as male rats developed kidney impairment faster than their female littermates [96,97].

The effects of hormone replacement therapy on kidney function remain inconsistent. Some studies report increased microalbuminuria and reduced GFR following estrogen therapy, whereas others describe reduced albuminuria in postmenopausal women receiving hormone replacement therapy [98,99,100]. Data from individuals receiving gender-affirming hormone therapy further support differential hormonal effects on kidney physiology, with estradiol generally showing protective effects and testosterone exerting opposing effects in humans [60]. In addition, gender-affirming hormone therapy has shown to increase serum creatinine levels in transgender men while in transgender women the levels are not affected [101].

Altogether, these lines of evidence highlight that sex hormones have complex and likely sex- and dose-dependent effects on kidney health [60,102].

### 2.3. Sex Hormones and Their Influence on Organ Crosstalk in CKD

Sex steroid hormones contribute to physiological differences between males and females across multiple organ systems through the widespread expression of their receptors in tissues such as the kidney, gut, liver, and brain [64,103,104,105]. This is particularly relevant in CKD, which is increasingly recognized as a systemic disorder involving bidirectional communication between the kidneys and other organs, as discussed below in Section 3.3.

Clinical studies show that men generally experience faster kidney function decline, higher all-cause and CV mortality rates, more frequent bone alterations, and a higher lifetime risk of kidney failure compared with women [60,85]. The lower CKD incidence and progression observed in premenopausal women compared with age-matched men diminishes after menopause, further supporting that estrogens play a key protective role [35,36,52,106,107,108,109]. Reduced testosterone levels are frequently observed in men across all stages of CKD and have been identified as an independent predictor of increased mortality [110]. Although the precise mechanisms underlying this association remain unclear, reduced testosterone levels have been linked to several major CKD comorbidities, including atherosclerosis, CKD-mineral bone disorder (CKD-MBD), metabolic syndrome, CVD, and systemic inflammation [111,112,113,114]. In contrast, elevated androgen levels in women are linked to obesity and CVD [115,116].

Sex hormones also influence the bone-vascular axis, which is strongly disrupted in CKD-MBD, a condition closely associated with vascular calcification (VC) and increased CV morbidity [85,117]. Testosterone stimulates bone formation through effects on osteoblasts and osteoclasts, but testosterone deficiency in hypogonadal men with CKD contributes to impaired bone turnover [113,118]. At the vascular level, testosterone has been associated with increased VC, whereas estradiol appears protective [119,120].

Another important mediator of inter-organ communication in CKD is the gut microbiota. Gut dysbiosis contributes to CKD progression and CKD-MBD, and evidence suggests that sex hormones partly regulate these effects [121]. Germ-free mice are protected from bone loss induced by sex steroid deprivation, indicating a role for the gut microbiome in estrogen-related bone metabolism [122]. In CKD mouse models, sex- and age-dependent differences in microbial composition have also been linked to higher levels of UTs such as trimethylamine N-oxide (TMAO) and indoxyl sulfate (IxS), as well as increased burden of cerebral microhemorrhages [123].

In parallel, CKD itself disrupts endocrine homeostasis. Men with CKD frequently develop hypogonadism with reduced total and free testosterone levels, whereas estradiol levels in women appear less affected in CKD compared to controls [124]. The uremic milieu may impair the hypothalamic-pituitary-gonadal axis, reducing hormone synthesis while chronic inflammation and nutritional deficiencies further suppress gonadal function [113]. Thus, CKD both alters sex hormone levels and is influenced by them.

Overall, sex hormones interact with multiple pathogenic pathways in CKD, including RAAS activation, inflammation, vascular calcification, bone metabolism, and gut dysbiosis. These processes may partially explain sex differences in disease trajectories, as well as why hormonal status, e.g., menopause and hypogonadism, can alter CKD risk and progression. As we will discuss in the following section, sex hormones may regulate several key determinants of UT biology, including gut microbiota composition, hepatic metabolism, plasma protein binding, and membrane transporter expression. Through these mechanisms, hormonal status could directly influence toxin generation, systemic exposure, and tissue susceptibility to toxin-induced injury. Conversely, CKD-associated endocrine dysfunction may further impair these regulatory mechanisms, contributing to sex differences in uremic toxicity and disease progression.

## 3. Sex Differences in UT Biology

UTs originate from diverse metabolic pathways and differ in biochemical properties, biological activity, and systemic effects. Increasing evidence, mainly from animal studies, suggests that biological sex and sex hormones, modulates key processes involved in UT biology, including their generation, metabolism, transport, and clearance [32,50,79]. In addition, sex differences in diet, gut microbiota, metabolic capacity, and kidney function may further affect systemic toxin exposure. In the following sections, we discuss UT classification and origin, sex-specific UT profiles, and the role of sex hormones in the inter-organ processes governing toxin generation, metabolism, and toxicity.

### 3.1. Classification and Origin of UTs

As proposed by the European Uremic Toxin Work Group (EUTox), UTs are traditionally classified based on molecular weight (MW) and degree of protein binding, two properties that strongly influence dialytic removal [10,125,126,127]. This framework distinguishes (i) small water-soluble compounds, MW < 500 Da (e.g., urea and creatinine), (ii) larger middle molecules, mostly peptides MW > 500 Da (e.g., β2-microglobulin or B2M, which are less efficiently removed by conventional dialysis) and (iii) protein-bound UTs (PBUTs), most of which have a low MW < 500 Da that bind reversibly to plasma proteins, thereby limiting their removal by dialysis [8,45,128]. Importantly, UTs represent a highly heterogeneous group of compounds with diverse biochemical properties and biological effects [45,127]. PBUTs, such as IxS and *p*-cresyl sulfate (pCS) are of particular clinical interest because of their poor clearance and strong associations with CKD complications such as fibrosis and CV disease [11,27,129,130].

Although this classification is important for understanding extracorporeal removal strategies, increasing attention has shifted toward classifying UTs according to their origin, as this may provide mechanistic and therapeutic insights [131]. UTs may originate from exogenous intake, endogenous host metabolism, and/or gut microbial metabolism [8]. For example, oxalate originates from both dietary intake (exogenous) and endogenous metabolism, whereas many PBUT precursors are generated through intestinal microbial activity [11]. Focusing on toxin origin provides important mechanistic insights and may open new preventive and therapeutic opportunities aimed at delaying CKD progression and reducing CV risk, particularly at the earlier stages of disease [8,126,132]. In the following sections, we discuss current evidence for sex-specific UT profiles and how biological sex and sex hormones may influence the multi-organ processes underlying UT generation, metabolism, and toxicity in CKD.

### 3.2. Sex-Specific UT Profiles: What Do We Know?

In clinical studies, levels of various UTs were higher in men, while symptoms were reported to be more frequent in women with advanced CKD [24]. This evidence indicates that UT plasma profiles per se do not explain sex differences in clinical presentation. In the general population, circulating levels of pCS and IxS are higher in men than in women, with sex, age, and kidney function identified as independent determinants of toxin concentrations [133]. In CKD patients, both pCS and IxS increase progressively with declining eGFR [134,135]. While these differences may partly reflect sex-specific gut microbiota composition, alterations in intestinal absorption, metabolism, and kidney clearance may also contribute, although human evidence is lacking so far [136].

Because sex-stratified human data remain scarce, animal models provide important mechanistic insights into sex-specific UT profiles. State-of-the-art preclinical CKD studies offer important translational context for clinical observations and currently provide most of our mechanistic insight into sex-specific toxin profiles and their consequences. Experimental studies comparing adenine-induced CKD and nephrectomy (Nx) models show that sex significantly influences circulating PBUT levels and their systemic consequences [17,123,137]. Despite similar degrees of kidney dysfunction, these models display distinct toxin patterns, suggesting that toxin accumulation is determined not only by GFR decline but also by sex-dependent differences in toxin generation, metabolism, and clearance. More specifically, in adenine-induced CKD mouse models, both sexes show increased circulating TMAO and IxS levels, whereas pCS elevation is more pronounced in Nx mouse models [17,137]. Female Nx mice exhibit higher TMAO and IxS concentrations than males, while indole-3-acetic acid (IAA) increases selectively in male adenine-fed mice. These findings suggest that hormonal regulation, microbial metabolism, and transporter activity may all contribute to sex-specific toxin profiles. Importantly, sex differences extend beyond circulating toxin levels. Male CKD mice develop more pronounced muscle wasting and alterations in cardiac signaling pathways, whereas females appear relatively protected from these complications, indicating that hormonal influences may modulate tissue susceptibility to uremic toxicity.

Animal models only partially reproduce human CKD [138,139,140]. Despite this, experimental studies consistently show female protection from kidney injury and sex-dependent molecular responses in both rodent and pig models, broadly paralleling the lower CKD susceptibility observed in women, while specific toxin patterns (e.g., higher PBUT levels in female Nx mice) may diverge from human UT profiles and should therefore be interpreted with caution [32,86,141]. Together, these data suggest that biological sex influences both systemic toxin exposure and the biological response to UTs, meaning that similar toxin concentrations may have different pathological consequences depending on hormonal and physiological context. It is essential to consider both the potentially harmful and beneficial effects of individual UTs, as well as how these effects may differ between sexes. Within the next section, we discuss how sex differences may arise at the levels of UT generation, metabolism, and toxicity.

### 3.3. The Role of Sex in UT Generation and Metabolism: Inter-Organ and Inter-Organism Crosstalk

Under physiological conditions, gut microbial metabolism supports host homeostasis [1,142]. In CKD, however, the accumulation of microbial metabolites shifts their role from signaling to pathological toxicity [126,143,144]. This systemic exposure is governed by the Remote Sensing and Signaling Theory (RSST), which describes an interacting network of transporters such as organic anion transporter 1 (OAT1) and drug-metabolizing enzymes that facilitate communication across the gut–liver–kidney axis [49,126,130,142]. Key toxins such as IxS, pCS, IAA, *p*-cresyl glucuronide (pCG), and TMAO originate from dietary proteins processed by the microbiota into precursors like indole and *p*-cresol [11,131,142,145]. These metabolites are subsequently processed in the liver by phase I and II enzymes (sulfation and glucuronidation) before elimination by the kidneys [146,147]. Notably, while contributing to uremic toxicity [145,148], these pathways also produce beneficial molecules such as the neuroprotective indole-3-propionic acid [149,150,151], highlighting that these metabolic routes are not uniformly harmful.

The gut–kidney axis functions bidirectionally: impaired kidney function triggers gut dysbiosis through ammonia accumulation and altered pH, which in turn accelerates CKD progression [152,153]. Recent evidence further indicates that gut microbiota composition is shaped not only by kidney dysfunction but also by factors such as diet, medication use, and intestinal transit time [154]. Dysbiosis can impair intestinal barrier integrity [155,156,157], allowing bacterial products to enter the circulation and trigger chronic inflammation via cytokines such as interleukin-6 (IL-6) and tumor necrosis factor-α (TNF-α) [46,47]. This inflammatory state contributes to kidney damage and cardiovascular complications, [152,155,156,157], highlighting the tight integration of metabolic and immune pathways within the gut–liver–kidney axis. [46,47].

Sexual dimorphism can occur at multiple levels of the gut–liver–kidney axis and may influence UT generation, metabolism, protein binding, inflammatory signaling, and kidney clearance. Below, we summarize sex differences in key components along this axis, highlighting their potential relevance for sex-dependent uremic toxicity and the role of sex hormones in mediating at least part of these differences.

#### 3.3.1. The Gut and Its Microbiome

Sex is an important determinant of gut microbiome composition and functional capacity, thereby influencing UT precursor generation and metabolism [158]. Large multicenter metagenomic studies in humans demonstrate clear sexual dimorphism in gut microbial communities [52]. For instance, *Dorea*, a genus associated with TMAO production, is positively associated with testosterone, whereas *Slackia* shows a strong association with circulating estradiol levels [58].

In healthy women, the gut microbiome changes across reproductive life stages [58,159]. Contrary to this, the microbiome from postmenopausal women gradually resembles that of men, suggesting sex hormones play a key role in maintaining microbial homeostasis. Animal studies further support this, showing that ovariectomy induces microbial dysbiosis, whereas in humans it is associated with an increased abundance of a specific taxa, including *Clostridium bolteae* [160,161,162]. Estrogens and their metabolites influence microbiota composition directly or indirectly [52,54]. For example, ER activation promotes an intestinal environment less permissive to pathogenic taxa, whereas ER inhibition or deficiency promotes dysbiosis [55]. Notably, female-associated microbiomes are often characterized by stronger anti-inflammatory and renoprotective features [52]. In contrast, elevated androgen levels are linked to reduced microbial diversity and dysbiosis in certain clinical contexts (e.g., polyendocrine metabolic ovarian syndrome), although effects may vary depending on metabolic context [50]. Given that many PBUTs, such as IxS, pCS and TMAO, originate from gut microbial metabolism, sex-hormone mediated microbiome differences may contribute to sex-specific systemic toxin exposure and CKD outcomes.

In humans, identifying sex-related microbiome differences is complicated by multiple confounders, including age, diet, medication use, body composition, and intestinal transit time, many of which also exhibit sex-specific patterns [158,163]. Although sex-specific gut dysbiosis has been reported in CKD patients, its direct impact on UT generation has not yet been quantified [164]. Additionally, considerable interindividual variability in IxS production has been observed, but no sex-stratified analyses were performed [165]. Similarly, patients initiating peritoneal dialysis exhibit distinct UT profiles linked to differences in microbial diversity, yet without sex-based stratification [166]. Therefore, further studies incorporating sex-stratified analyses are essential to elucidate the role of sex in gut-derived UT generation.

#### 3.3.2. Liver Metabolism

The liver is a central processing hub in UT metabolism, and hepatic physiology exhibits profound sexual dimorphism [146,147], including sex differences in metabolic enzyme expression and activity [167]. Enzymes implicated in the biotransformation of UT precursors such as cytochrome P450 enzymes (CYP450s), which are phase I enzymes and play a role in the hepatic metabolism of indole and *p*-cresol [168,169]. In vitro studies show that CYP1A2, CYP2D6, and CYP2E1 are the most active enzymes that mediate the bioactivation of *p*-cresol. Interestingly, these enzymes display sex differences in activity in humans, with general trends of CYP1A2 activity being higher in men while CYP2D6 activity is reported to be higher in women [169,170]. In the case of CYP2E1, animal data show overall higher messenger ribonucleic acid (mRNA) expression levels in females, while there are no significant differences in human livers [170].

Expression patterns of genes encoding uridine diphosphate (UDP)-glucuronosyltransferases (UGTs) and sulfotransferases (SULTs), two enzyme families also involved in UT handling, show differences between males and females [171]. In humans, UGT2B28, UGT2A3 and UGT2B10 are more highly expressed in females, while UGT2B17 shows higher expression levels in male liver tissue. UGT1A6, the main enzyme responsible for pCG formation in humans, is primarily expressed in the liver, with reported sex differences in pigs but not consistently in rodents or humans [172,173,174,175].

As an example of sulfation, sulfate transfer during IxS production is mediated by SULT1A1 [176,177]. Hepatic SULT1A1 expression decreases in response to IxS accumulation, suggesting feedback regulation [14]. In rats, SULT1A1 mRNA levels are higher in males than females, whereas in mice exposed to IxS, females show increased SULT1A1 expression but reduced CYP450 activity compared with males [3,178]. Moreover, sulfation capacity is influenced by host-microbiome interactions, including sulfur metabolism pathways that are linked to microbiota compositions and function [179,180]. Together, these findings highlight potential, but species- and context-dependent, sex differences in hepatic toxin processing.

#### 3.3.3. Plasma Protein Binding

Sex differences in UT plasma levels may also relate to differences in plasma protein binding. PBUTs account for approximately 25% of identified UTs and primarily bind to human serum albumin (HSA), the most abundant plasma protein [181,182]. In patients with advanced CKD, albumin transport capacity is reduced, and higher unbound toxin fractions are associated with CKD-related complications [183]. This is particularly relevant for PBUTs such as pCS and IxS, which are highly albumin-bound (approximately 90% and 93%, respectively) and therefore poorly removed by dialysis [184].

Sex hormones may influence HSA concentrations and binding dynamics, thereby affecting PBUT bioavailability and toxicity [59,185,186]. Women generally exhibit lower HSA levels than men, and albumin concentrations decline more rapidly with age in women until they approximate male levels around the age of 60 [185,186]. It is also reported that the binding of sex hormones to albumin happens in a nonlinear manner, with differences in binding dynamics in low versus high concentrations of the protein [59]. Because sex hormones themselves circulate partly bound to albumin and SHBG, competition for albumin binding sites between hormones and PBUTs may occur [187]. Such interactions could alter the free fractions of both hormones and toxins, thereby influencing toxin distribution, biological activity, and kidney clearance. In addition, CKD-associated oxidative stress can modify HSA structure and binding affinity, further affecting PBUT binding properties [182,188]. Together, these observations suggest that sex-dependent differences in albumin levels and binding dynamics may contribute to variation in PBUT bioavailability and uremic toxicity.

#### 3.3.4. Kidney Clearance

Sex-related differences in kidney clearance are primarily driven by differences in kidney size and morphology. Men generally have larger kidneys, glomerular mass, and total glomerular volume than women, although the total number of glomeruli is similar between sexes [32,189]. These structural differences contribute to a higher baseline GFR and greater kidney workload in men, which may partly explain their faster CKD progression [52]. Although eGFR values in CKD are often comparable between sexes, women on dialysis are less likely to experience a 50% decline in eGFR, even after adjustment for confounding factors [190,191].

Sex differences also exist in the expression of membrane transporters and metabolic enzymes in tubular epithelial cells, which are central to UT handling. Animal studies show sex-specific expression patterns of apical and basolateral transporters, affecting urinary composition and toxin secretion [41]. Male rats generally exhibit higher expression of secretory transporters and lower expression of reabsorptive transporters, whereas the opposite pattern is observed in females [67]. The role of these transporters in UT handling and CKD will be discussed further in Section 3.4.2.

In addition to transporters, kidney tubular cells contribute to toxin metabolism through enzymes such as UGTs, which exhibit the highest activity in the kidney (apart from the liver) [192]. Notably, in vitro studies show that UTs such as IxS and IAA can inhibit UGT activity in human kidney cells, potentially impairing kidney metabolic capacity in CKD, although in vivo relevance remains to be fully established [192,193]. Furthermore, in vivo studies demonstrate that sex differences in specific kidney UGT mRNA expressions are regulated by androgens, suggesting hormonal regulation of sex-specific toxin metabolism [194].

#### 3.3.5. Immune-Mediated Mechanisms

Immune signaling within the gut–kidney axis also shows sex bias [46,47]. In CKD, UTs such as UA stimulate inflammatory pathways involving interleukin-1β (IL-1β) and TNF-α, promoting tubular injury, oxidative stress, and fibrosis, which further accelerates toxin accumulation [195]. Notably, TNF-α and IL-1β levels are higher in male than in female kidney tissue in patients with CKD, with these sex differences closely linked to sex hormone regulation [52]. Such immune dimorphism may therefore modify susceptibility to toxin-induced kidney injury.

Overall, crosstalk between organisms, organs and molecular pathways is fundamental within the gut–kidney axis. In the context of uremic toxicity and CKD, where homeostasis is disrupted, it is important to recognize that sex differences may exist at multiple levels along this axis. These differences may shape inter-organism and inter-organ communications, thereby influencing uremic toxicity, as will be detailed in the next section.

### 3.4. Sex Differences in Uremic Toxicity

#### 3.4.1. Multi-Organ Toxicity and the Role of Sex

Uremic toxicity is inherently sex-modulated, as biological sex influences both systemic UT exposure (generation, protein binding, clearance) and tissue susceptibility to their downstream signaling effects. Accumulation of UTs contributes to uremic syndrome, which involves kidney, gastrointestinal, neurological, CV, immune, and skeletal complications [128,184,196,197,198]. Clinical manifestations differ between sexes and across life stages such as puberty, pregnancy, menopause, and aging, suggesting that sex-dependent biology shapes toxin-related pathophysiology beyond toxin concentrations alone [24,41,42]. The multi-organ toxicity of UTs is discussed in the following sections, together with evidence supporting sex differences in toxin levels, signaling pathways, and clinical outcomes.

##### Nephrotoxicity

UTs contribute to kidney injury mainly through oxidative stress, inflammation, and mitochondrial dysfunction [184,199,200]. IxS, for example, targets PTCs, activates the nuclear factor kappa B (NF-κB) pathway, triggers pro-inflammatory cytokine release, and accelerates cellular senescence [201,202]. Additionally, pCS contributes to nephrotoxicity by stimulating reactive oxygen species (ROS) production [203,204], impairing mitochondrial function [192], and disrupting efflux transporter activity, ultimately promoting apoptosis [205].

Sex-dependent biology likely modifies both toxin exposure and injury responses in the kidney. Rodent studies indicate that males generally produce more ROS and experience greater oxidative damage, particularly in the context of kidney injury [108]. Furthermore, sex differences in mitochondrial homeostasis are increasingly recognized as determinants of kidney injury susceptibility [206]. In line with this, male CKD animals develop more severe tubular dilation, interstitial fibrosis, and inflammation, whereas females show attenuated tubular injury and fibrosis [207]. Together, these findings suggest that UT-induced nephrotoxicity involves shared pathological pathways (ROS, inflammation, mitochondrial dysfunction) but differs in severity and progression between sexes.

##### Cardiotoxicity

CV uremic toxicity results from the combined effects of toxins on oxidative stress, endothelial dysfunction, and vascular calcification (VC) [16]. UA, IxS and pCS stimulate inflammatory responses, RAAS activation, and fibrosis, contributing to uremic cardiomyopathy [208,209,210,211]. Furthermore, compounds such as asymmetric dimethylarginine (ADMA) impair endothelial function by disrupting NO metabolism [212], while gut-derived toxins (IxS, pCS, TMAO) directly promote VC through the activation of pro-inflammatory and coagulation pathways [18,213,214,215,216,217,218].

Sex modulates CV vulnerability to UTs through differences in hormonal milieu, NO biology, and calcification patterns. Women with CKD generally show lower risks of CV events and mortality than men, although this protective effect declines after menopause [89,190,219]. Sex differences in NO metabolism may further contribute to differential vascular responses to UTs. Women typically exhibit higher systemic NO production, whereas renal vascular tone in men appears more dependent on NO signaling [212,220,221]. Consequently, UT-induced disruption of NO pathways may have sex-specific vascular effects [222]. There are also sex differences in VC, with males having a tendency to acquire vascular and aortic valve calcification earlier in life, and females developing calcification post-menopause [119,223]. In addition, CKD mouse models demonstrate sex differences in circulating PBUT levels relevant to CV complications, such as TMAO and IxS [17]. Female nephrectomized mice, for example, show higher serum TMAO and IxS levels than males. Together, these findings indicate that both UT exposure and CV response are sex-sensitive, potentially contributing to sex differences in CKD-related CV outcomes.

##### Musculoskeletal Toxicity

UT accumulation impairs musculoskeletal health by disrupting bone turnover and promoting skeletal muscle wasting [224]. Toxins like kynurenine are associated with CKD-MBD [216,225,226], while PBUTs exacerbate muscle catabolism through oxidative stress, inflammation, and myostatin activation [227,228,229]. Specifically, IxS and IAA impair mitochondrial function in skeletal muscle, contributing to fatigue, weakness, and atrophy [24,230].

Sex can modify these outcomes through hormone-dependent regulation of bone turnover and differences in body composition. Structural and functional sexual dimorphism in the musculoskeletal system is well established, including differences in muscle mass, fiber size, and contractile properties [231]. Women generally have a higher body fat percentage and lower lean muscle mass compared to men [232]. In CKD, men experience more pronounced relative losses in muscle mass and strength, whereas bone alterations become more pronounced in women after menopause [233,234,235]. Hypogonadal ESKD women experience a higher bone turnover and more significant bone mass decrements [114,231]. Together, these findings suggest that UT toxicity might act on a background of pre-existing sex differences in bone and muscle biology, which are further modified by hormonal status during CKD progression.

##### Neurotoxicity

The central nervous system (CNS) is highly susceptible to uremic toxicity, particularly when the blood–brain barrier (BBB) is impaired [21]. Small water-soluble toxins, such as guanidine compounds, and a range of middle molecules may contribute to neurodegeneration [19,45,236,237]. Furthermore, the accumulation of PBUTs like IxS and pCS in brain tissue is linked to oxidative stress and neuroinflammation, resulting in cognitive impairment, memory loss, and uremic encephalopathy [45,128,238].

Sex may modulate CNS vulnerability via differences in BBB properties and protein-aggregation biology, potentially contributing to sex differences in symptom burden. In vitro data suggest sex differences in BBB strength, although the extent to which these findings translate to the in vivo human setting remains to be determined [239]. Sex differences in neurotoxic side effects of CKD have not been directly reported [240]. However, sex differences in uremic symptom burden, including cognitive symptoms, suggest possible sex-dependent CNS effects, but that warrants further investigation [24,42].

Altogether, uremic toxicity affects multiple organ systems and involves shared pathways such as oxidative stress and inflammation. The underlying pathogenic mechanisms, influenced by different UTs and shaped by sex, are complex and multifactorial. Indeed, these processes rely on intricate signaling networks that communicate across tissues, shifting normal physiological responses toward pathological states [14]. Many UTs exert cellular toxicity via specific membrane transporters, some of which may be influenced by sex, as discussed in the next section.

#### 3.4.2. Sex Differences in Membrane Transporter Proteins

Transporter-mediated tubular secretion is a central determinant of UT exposure, and sex-dependent transporter expression provides a mechanistic link between biological sex and uremic toxicity. CKD progression involves not only reduced GFR, but also progressive loss of proximal tubular function [241]. PTCs express transporters required for active secretion of uremic solutes into urine, while unbound toxins are cleared by glomerular filtration [11,125,242]. As CKD progresses, kidney transporter expression and activity decline, reducing clearance of PBUTs and increasing systemic exposure [125,242]. Crucially, these transport systems are not sex-neutral: multiple transporter families show sex-dependent expression at baseline and are hormonally regulated, implying that CKD-associated transporter dysfunction may produce different toxin profiles and intracellular exposures in males and females [66,243,244].

In addition, uremic solutes can inhibit or compete for transporter activity, creating feedback loops that further amplify toxin retention and alter drug handling [192,245]. Key transporter families involved include OATs, organic anion-transporting polypeptides (OATPs), multidrug resistance-associated proteins (MRPs) and other ATP-binding cassette (ABC) transporters, like ABCG2 [49].

**Figure 1:**
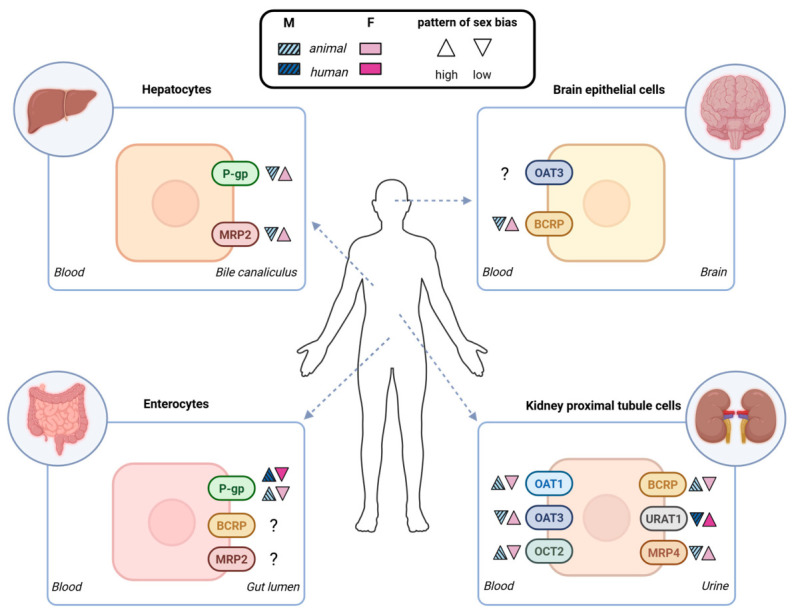
**Sex-biased expression patterns of key cell membrane transporters that mediate the transport of uremic toxins across several tissues relevant in CKD-uremic toxicity**. Schematic, partly based on [182], illustrating the sex bias in expression in hepatocytes, enterocytes, brain epithelium, and kidney proximal tubule cells of organic anion transporters 1/3 (OAT1/3), organic cation transporter 2 (OCT2), urate transporter 1 (URAT1), multidrug resistance-associated proteins 2/4 (MRP2/4), breast cancer resistance protein (BCRP), plus P-glycoprotein (P-gp). Specifically, male bias (M, blue triangles with lines), female bias (F, pink triangles), or unknown sex bias (question mark) are indicated, and the orientation of the triangles (up vs. down) indicates the direction of the sex bias. Data are synthesized from animal (light colors) and human (dark colors) studies. Created in Biorender. Oriana Nobus (2026) https://BioRender.com (accessed on 29 March 2026).

In this section, we examine the organ-specific expression of membrane transporters, their modulation by UTs, changes during CKD progression, and how biological sex might influence these interactions (Figure 1). Of note, depending on the study transporter expression may refer to transcriptional (mRNA) or translational (protein) levels, as specified. Regarding sex differences in transporter activity, evidence arises from in vitro (cell-based or molecular) and/or in vivo (animal or human) methods designed to measure the movement of specific substrates across epithelial barriers.

##### Kidney

In the kidney, active elimination of PBUTs depends on transporter-mediated tubular secretion, amongst others via OAT1/OAT3 [49,192,205,245,246], organic cation transporter 2 (OCT2) [182] and urate transporter 1 (URAT1) [247]. CKD triggers a progressive decline in the expression and function of these kidney transporters, which impairs toxin clearance and alters the metabolic capacity of the kidney [192,245]. Furthermore, uremic solutes such as IxS and hippuric acid (HA) create pathological feedback loops by inhibiting the activity of transporters including BCRP, and MRP4 [193]. UTs may also be involved in CKD progression by negatively affecting kidney tubule cell phenotype and functionality, through altering the expression of key tubular transporters thereby exacerbating secretion deficits as CKD progresses [205].

Sex differences in kidney tubular transporter expression emerge after puberty, indicating hormonal regulation [53]. An increasing body of research suggests that sexual dimorphism in kidney structure and function may be linked to sex-biased expression and activity of transporter proteins in the basolateral and apical membranes of tubular cells [41,76]. Specifically, high mRNA expression of secretory transporters (OAT1 and OAT3) was found in male rats, while mRNA levels of reabsorptive transporters (OAT2 and OAT5) were lower [66]. In contrast, female rats exhibited the inverse pattern, with low mRNA levels of secretory transporters and high levels of the reabsorptive transporters. In rat models, androgens stimulate the transcriptional expression of OAT1 and OAT3, whereas estrogens exert inhibitory effects [53,66,248]. Notably, not all studies concur as it was also reported that mRNA levels of OAT1 and OAT3 were respectively lower and higher in female compared to male mice [3]. Furthermore, protein expression data do not always reflect the mRNA patterns, as evidence also indicates that OAT3 protein expression was lower in male mice, whereas OAT1 protein expression was higher [243]. While findings on OAT3 remain inconsistent, the overall body of evidence suggests that kidney OAT1 expression is, at least in part, androgen-regulated [249]. OCT2 expression, both at the mRNA and protein level, is also typically higher in male rats and is positively regulated by testosterone [66,250,251]. Testosterone positively regulates OCT2 mRNA expression in mice, suggesting broader androgenic effects on tubular secretion pathways [252]. Estrogen regulation appears more complex and transporter specific. Both MRP3 and MRP4 tend to have higher expression at the mRNA and protein level in females [103,253]. For BCRP, gonadectomy increased BCRP transcriptional expression in female mice kidneys but did not affect BCRP mRNA levels in males, suggesting that sex hormones may regulate these sex-specific expression patterns [254]. In addition, estrogens have been reported to downregulate the secretory transporter BCRP, which contributes to UA secretion, while simultaneously suppressing the reabsorptive transporter URAT1 [255,256,257]. In humans, URAT1 is significantly more expressed at the transcriptional level in women compared to men [39,41]. However, data on sex-dependent kidney transporter expression in humans remain limited [76]. Despite species differences, partial conservation of transporter regulation supports the relevance of animal models for studying molecular mechanisms underlying sex differences in kidney physiology and disease [258].

Together, these findings indicate that sex hormones do not uniformly stimulate or inhibit kidney transport processes. These data also support a working model in which sex-dependent transporter expression and regulation can alter the cellular ‘dose’ of PBUTs in proximal tubules and could thereby modulate nephrotoxicity and systemic exposure, even at comparable eGFR. Importantly, emerging evidence suggests that sex differences in transporter expression are not limited to the kidney, but may also occur in other tissues, as will be detailed in the next sections.

##### Gut

Intestinal transporter remodeling in CKD impacts systemic toxin exposure by altering absorption and efflux of microbial metabolites [128,259]. In uremic conditions key efflux transporters, including P-glycoprotein (P-gp) and MRP2, are consistently downregulated, while effects on BCRP remain variable [260,261,262,263,264]. These alterations may facilitate the translocation of UT precursors into the portal circulation while simultaneously reshaping drug-toxin competition at shared transport sites [128].

Sex differences may also affect efflux transporters in the gut. P-gp expression is generally higher in males than females in both human and rodent studies [265,266]. In contrast, evidence for intrinsic sex differences in intestinal BCRP expression remains inconsistent and appears to be context-dependent. Several datasets suggest intestinal BCRP mRNA expression is not strongly sex-dependent in rodents and humans [254,267]. Yet, dietary interventions in rats, can regulate BCRP and MRP2 protein levels as well as hormonal plasma concentrations in the intestinal tract in a sex-dependent manner, implying that sex effects may emerge indirectly through diet, microbiome and hormonal interactions [268].

##### Liver

In the liver, the uremic milieu reprograms membrane transporter expression, altering hepatic export and drug disposition [170]. CKD is associated with organ-specific remodeling, with intestinal MRP2 decreasing while hepatic MRP2 increases, indicating adaptive but heterogeneous transporter responses to uremia [182,244,269]. Additionally, IxS can directly modify hepatic transport by increasing the expression and activity of P-gp, potentially influencing the pharmacokinetics of both endogenous solutes and drugs [270].

Similar to the kidney, hepatic MRP expression displays a female bias [244]. For example, female rat livers show higher MRP2 expression at both mRNA and protein levels [271] and mRNA levels of MRP4 are higher in female mice when submitted to a fasting period [103]. In addition, higher hepatic P-gp and MRP2 protein levels have been observed in female rats and are reduced by testosterone administration [272]. Altogether, these sex differences in membrane transporter proteins in the liver could potentially influence hepatic export of conjugated metabolites (including UTs), systemic exposure, and drug-toxin interactions in CKD.

##### Nervous System

At the BBB, transporter expression may act as a key gatekeeper for brain exposure to PBUTs. Since IxS and pCS are present in the brain in the same chemical form as in the kidneys, transporters involved in their handling across PTC (e.g., OAT3 and BCRP) may also mediate their transport across the BBB [273], although direct evidence is lacking [249,274].

In vivo preclinical data show no sex differences in OAT3 mRNA levels in brains of rats [275], whereas higher BCRP mRNA expression was found in the brains of female mice compared with males [254]. Even modest differences in BBB efflux may become relevant in CKD, where circulating PBUT levels are elevated and barrier integrity may be impaired.

Overall, sex differences in membrane transporter expression and regulation across kidney, gut, liver, and brain could substantially alter intracellular ‘dose’ and organ exposure to UTs, even when plasma concentrations and eGFR appear similar between sexes.

#### 3.4.3. UTs as Ligands and Signaling Molecules and the Role of Sex

Beyond passive accumulation, many UTs function as signaling molecules, altering gene expression and cellular phenotype after transporter-mediated uptake. This signaling function provides a mechanistic explanation for the multi-organ effects of uremic toxicity and helps to explain why toxicity depends not only on circulating concentrations but also on tissue-specific transporter expression and receptor availability [49,182].

In CKD, elevated levels of uremic tryptophan catabolites, especially IxS, may lead to dysregulation of aryl hydrocarbon receptor (AhR) activity, promoting vascular disease, kidney tissue fibrosis, and inflammation [270,276]. AhR is a ligand-activated transcription factor expressed in epithelial, endothelial, and immune cells and represents a key mediator linking toxin accumulation to tissue injury [277,278]. Aberrant AhR activation by UTs contributes to endothelial dysfunction and procoagulant effects, underlying much of IxS-induced CV toxicity [279,280,281,282,283,284]. In the CNS, AhR activation by IxS increases BBB permeability and has been associated with cognitive dysfunction [14,285]. Patients with ESKD, showing elevated levels of IxS, show increased BBB permeability compared to healthy controls (no sex-stratified analysis) [286]. Similarly, IAA induces endothelial oxidative stress and inflammation through AhR activation [280].

Sex-dependent regulation of AhR signaling may contribute to differential susceptibility to uremic toxicity [74]. In mice, basal kidney AhR mRNA expression is higher in females than in males and correlates with CKD severity. Upon Nx, females showed a decrease in AhR mRNA transcripts while the expression was unaffected in males. AhR also interacts directly with estrogen and androgen receptor pathways through transcriptional crosstalk and shared co-regulators [287]. This bidirectional interaction suggests that sex hormones may influence cellular responses to UTs, while toxin-mediated AhR activation may in turn alter hormone signaling and tissue susceptibility.

Overall, in addition to their direct effects on the kidney, sex differences may modulate multiple critical components of the gut–liver–kidney axis. Tissue-specific expression of membrane transporters and receptor availability determine the accumulation and cellular effects of UTs in different organs, thereby shaping organ vulnerability. In addition, sex differences may regulation gut microbial composition, plasma protein binding, transporter expression, and receptor signaling. Together, these mechanisms influence UT generation, distribution, cellular uptake, and clearance, providing framework linking hormonal status (e.g., aging, menopause, and CKD-associated hypogonadism) to sex-dependent differences in uremic toxicity.

## 4. Clinical Considerations of Sex-Dependent Uremic Toxicity in CKD

While experimental and mechanistic studies indicate sex differences in the generation, metabolism, and biological effects of UTs, their direct clinical relevance remains incompletely established. Current clinical evidence is limited and often not specifically designed to evaluate sex differences. Therefore, the considerations below should be viewed as emerging perspectives rather than established practice recommendations.

Clinical studies suggest that UTs may contribute to morbidity and mortality in CKD [288,289]. However, interpretation of individual toxins remains complex. For example, higher circulating 3-carboxy-4-methyl-5-propyl-2-furanpropionate (CMPF) levels have been associated with lower mortality risk in older patients with advanced CKD (not stratified by sex), although whether this reflects direct biological effects or healthier dietary patterns remains uncertain [24,289].

Several PBUTs, including IxS and pCS, increase with declining kidney function and have been associated with adverse CV, neurological, and musculoskeletal outcomes [27,290,291,292,293,294,295,296]. Clinically, the UT burden is managed through treating the underlying CKD drivers, diet and gut-directed therapies and improving clearance (e.g., advanced dialysis strategies). However, with growing attention to the uremic toxicity in CKD context, it is essential to consider both the potentially harmful and beneficial effects of individual UTs, as well as how these effects may differ between sexes.

Because many PBUTs are cleared predominantly by tubular secretion, altered circulating levels may partly reflect impaired tubular transport capacity [28,29,291]. This may provide complementary information to filtration-based markers such as creatinine or eGFR, which mainly reflect glomerular function, and enhance the clinical management of CKD through earlier diagnosis, better risk stratification and more personalized treatment [297,298]. However, UT concentrations are additionally influenced by diet, microbiome composition, protein binding, toxin generation, and transporter activity. Since several of these factors may differ by sex, further studies are required before UTs can be considered reliable biomarkers of tubular dysfunction or before sex-specific interpretation strategies can be proposed.

UTs have also been implicated in CKD-related symptoms such as fatigue, pruritus, and cognitive complaints [42,299,300]. Women with CKD often report a greater symptom burden than men, although the underlying causes are likely multifactorial [24,42]. It remains possible that sex-related differences in toxin exposure, free toxin fractions, transporter activity, or tissue susceptibility contribute to these observations. However, direct clinical evidence linking sex-specific UT profiles to symptom burden is currently limited and inconsistent. Experimental data also suggest interactions between UTs and drug transport or metabolism pathways [3,270,293,294,295]. Whether these mechanisms translate into clinically meaningful sex-specific differences in pharmacotherapy remains insufficiently studied.

Overall, sex may contribute to variability in UT burden and its clinical manifestations, but dedicated sex-stratified clinical studies are needed before implications for risk prediction, dialysis prescription, or pharmacotherapy can be determined.

## 5. Research Gaps and Future Directions

Although substantial progress has been made in characterizing UTs, personalized treatment strategies for CKD remains limited [45]. Current approaches mainly rely on generalized toxin classification and clearance strategies that do not account for interindividual variability in toxin handling. Genetic background, gut microbiota composition, comorbidities, age, environmental factors and sex can all influence the production, metabolism, and toxicity of UTs.

As discussed in this review, growing evidence identifies sex as an important biological variable of uremic toxicity. Nevertheless, most therapeutic approaches do not adequately account for interindividual variability, including sex differences. Failure to incorporate these differences may limit the effectiveness of risk stratification and targeted interventions. The following sections therefore summarize current research gaps and discuss future directions aimed at improving understanding of sex-dependent determinants of uremic toxicity in CKD.

### 5.1. Improving Our Understanding of Sex-Dependent Determinants of Uremic Toxicity in CKD

UT levels and toxicity in CKD are determined by interconnected processes including toxin generation, metabolism, and clearance, many of which are sex-specific and partly regulated by sex hormones. In addition to hormonal effects, sex chromosomes, sex-specific genetics and epigenetics, organ hemodynamics, and anatomical differences may also contribute to sex-dependent uremic toxicity [52]. As these factors interact at multiple biological levels, disentangling their respective contributions in experimental and clinical settings remains challenging. Physiologically based computational and experimental models may help integrate these complex interactions, provide mechanistic insight, and improve translation between preclinical findings and clinical outcomes [301].

#### 5.1.1. Computational Modeling of Sex Differences in Uremic Toxicity

Compartmental models are computational frameworks used to quantitatively predict the absorption, distribution, metabolism, and excretion of compounds (e.g., UTs, drugs) within the human body. These models represent the body as interconnected compartments (e.g., gut, liver, kidney, brain) which are linked through a blood compartment. Mathematical equations (e.g., ordinary differential equations) describe the transfer of compounds (e.g., UTs) between compartments over time, enabling the simulation of concentration-time profiles in plasma and individual organs. These models integrate physiological parameters including organ size, blood flow, tissue composition, transporter expression, and metabolic activity derived from experimental and clinical data [302].

Physiologically based compartmental models are particularly valuable in CKD research, where impaired kidney clearance significantly alters UT and drug handling. Existing CKD models simulate disease-related changes in kidney blood flow, GFR, and transporter expression to study pharmacokinetics, biomarker development, and UT-drug interactions [303,304,305,306,307,308,309,310]. Note that few computational models explicitly incorporate UTs [308,311]. Computational approaches are also well suited to studying sex differences, as model parameters can be adapted to reflect sex-specific physiology, including organ volumes, GFR, and transporter expression [76]. For example, modeling studies from the Layton group demonstrated sex-specific differences in renal electrolyte transport, with greater distal tubular Na+ transport in females [80,312,313,314]. Integrating CKD-specific and sex-specific modeling approaches may therefore improve understanding of sex-dependent uremic toxicity and support the development of more personalized therapeutic strategies and optimized drug research.

#### 5.1.2. Sex-Aware Experimental Models to Study UT Handling

In addition to computational modeling, experimental systems that explicitly incorporate biological sex may help to generate the mechanistic and quantitative data needed to develop and validate sex-aware models of uremic toxicity [41,315]. In vitro models provide controlled environments in which toxin concentration, exposure duration, albumin binding, transporter expression, and hormonal conditions can be precisely manipulated [316,317,318]. Compared with in vivo or clinical studies, in vitro models allow direct investigation of intrinsic sex differences in toxin transport, accumulation, and toxicity while minimizing confounding factors such as age, comorbidities, medication use, and hormonal variability [41,315,317,319,320]. Primary human kidney PTCs are particularly relevant because they mediate transporter-dependent secretion of many UTs. Male and female-derived cells retain intrinsic sex-specific characteristics, including differences in transporter expression, metabolic activity, and cellular stress responses [41,319]. These systems enable mechanistic investigation of sex-dependent differences in transporter activity, including key transporters such as OAT1, OAT3, OCT2, and ABC transporters [41]. However, traditional cell lines often lose critical transporter expression (e.g., OAT1/3), limiting their use for physiological studies [321,322].

Kidney organoids derived from induced pluripotent stem cells provide complementary multicellular models that better recapitulate kidney tissue organization [323,324]. These systems also allow independent manipulation of hormonal conditions, enabling separation of intrinsic genetic sex effects from hormone-mediated regulation, which is particularly relevant in CKD-associated endocrine disturbances [36,320].

Bioengineered systems, including kidney-on-chip platforms and bioartificial kidney models, further improve physiological relevance by reproducing tubular architecture, fluid flow, and polarized transporter expression [325,326]. Incorporating cells from male and female donors into these systems enables direct investigation of sex-dependent differences in toxin handling that more closely resemble in vivo physiology, while maintaining strict experimental control [41,327].

Together, these experimental platforms provide quantitative data on sex-specific transporter activity, toxin uptake rates, and cellular responses, that can be integrated into physiologically based computational models. The integration of in vitro experimental systems with computational modeling represents a critical step toward sex-informed predictive models of UT handling and personalized therapeutic strategies in CKD.

### 5.2. Prospects for Future Research: Sex-Aware Uremic Toxicity in CKD

**Figure 2:**
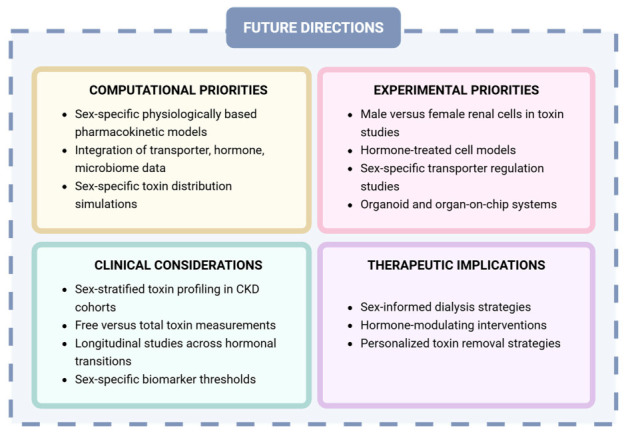
**Overview of future research directions to improve our understanding of sex-specific UT handling in CKD.** Priorities are categorized into computational modeling, experimental approaches, clinical considerations and therapeutic implications. Created in Biorender. Oriana Nobus (2026) https://BioRender.com (accessed on 29 March 2026).

Despite substantial progress in characterizing sex differences in UT biology, major gaps still limit translation into personalized medicine (Figure 2). Large, well-phenotyped human cohorts with systematic sex-stratified UT profiling are scarce, and most CKD studies either lack comprehensive toxin panels or do not perform sex-specific analyses. Although initiatives such as the EUTox consortium represent important steps forward, harmonized datasets incorporating free and total toxin concentrations, hormonal status, sex, age, CKD stage and longitudinal outcomes are still lacking. Sex-specific exposure-response relationships for key UTs remain undefined, and it is unclear whether equivalent toxin levels confer similar biological risk in males and females. Defining sex-specific dose–response curves will likely require integration of mechanistic in vitro studies, computational modeling, and sex-stratified clinical outcome analyses.

Life-stage-specific data is also lacking, particularly in adolescents and young adults, with hormonal status rarely being recorded. Longitudinal studies across hormonal transitions (e.g., pregnancy and menopause) are advised to determine whether sex differences in UT handling change across the lifespan.

In parallel, there is a lack of systematic information on sex-dependent transporter expression across kidney and extra-renal organs (liver, gut, brain) in both healthy and CKD contexts. The absence of systematic evaluation across hormonal states prevents validation of proposed differences in tubular secretion and PBUT clearance. Furthermore, few studies simultaneously assess microbiome composition, hepatic metabolism, circulating UT profiles, kidney handling, and sex hormone status. Without such multi-organ approaches, the upstream drivers of sex-specific toxin generation remain poorly defined.

Finally, current experimental and computational models largely assume sex-neutral parameters, limiting their ability to capture sex-dependent toxin kinetics, transport, and protein binding. Unfortunately, data gaps in sex-specific (kidney) pathophysiology limit accurate calibration of sex-specific parameters and model validation, leaving models reliant on extrapolations from male-dominated preclinical data or on pooling inconsistent sources.

Sex differences in UT handling and toxicity arise from interconnected processes regulated by sex hormones. These hormones influence gut microbiota composition, hepatic metabolism, transporter expression, and tissue susceptibility to toxic signaling. Consequently, similar circulating toxin levels may result in different intracellular exposure and biological responses between sexes. This framework may help explain sex differences in CKD progression, symptom burden, and complications despite comparable eGFR and toxin concentrations. Future work could therefore focus on developing integrated in vitro, in vivo, and clinical datasets of sex-specific UT toxicity.

## 6. Conclusions

**Figure 3:**
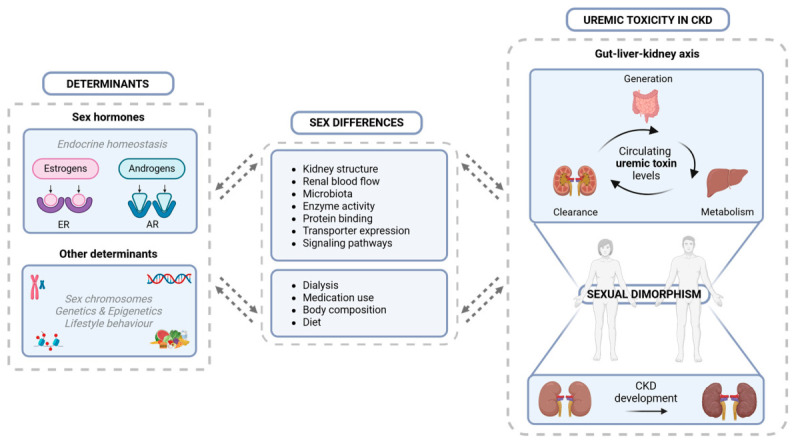
**Graphical abstract: sex-dependent determinants of uremic toxicity and CKD across multiple biological axes.** Schematic illustration of how sex differences in certain factors (e.g., microbiota and diet) modulate uremic toxin (UT) generation, gut–liver–kidney axis metabolism/clearance, and circulating UT levels, ultimately driving sexual dimorphism in CKD susceptibility and progression. Key determinants include sex hormones (estrogen/androgen-mediated), sex chromosomes, (epi)genetics, and lifestyle/behavioral factors. Created in Biorender. Oriana Nobus (2026) https://BioRender.com (accessed on 29 March 2026).

In conclusion, UTs are central mediators of CKD progression and its systemic complications. Growing evidence indicates that sex hormones regulate multiple determinants of uremic toxicity, including the gut microbiome, hepatic metabolism, transporter expression, protein binding, and downstream cellular signaling pathways, thereby likely contributing to sex differences in CKD pathophysiology (Figure 3). Through these mechanisms, biological sex influences both toxin exposure and tissue susceptibility, meaning that similar circulating toxin levels could potentially induce different biological effects in males and females. Despite these emerging insights, current clinical practice does not routinely account for sex differences in the interpretation of toxin levels or in therapeutic strategies targeting uremic toxicity, largely because supporting evidence remains limited. Further integration of sex-specific perspectives into research frameworks and prospective clinical studies may advance precision nephrology and determine whether the management of CKD-related complications can be improved.

## Data Availability

No new data were created or analyzed in this study.

## Abbreviations

The following abbreviations are used in this manuscript:

ABC	ATP-binding cassette
ADMA	Asymmetric dimethylarginine
AhR	Aryl hydrocarbon receptor
AR	Androgen receptor
B2M	β2-microglobulin
BBB	Blood–brain barrier
BCRP	Breast cancer resistance protein
CKD	Chronic kidney disease
CKD-MBD	Mineral bone disorder
CMPF	3-carboxy-4-methyl-5-propyl-2-furanpropionate
CNS	Central nervous system
CV	Cardiovascular
CVD	Cardiovascular disease
CYP450	Cytochrome P450
eGFR	Estimated glomerular filtration rate
ER	Estrogen receptor
ESKD	End-stage kidney disease
EUTox	European uremic toxin work group
HA	Hippuric acid
HSA	Human serum albumin
IAA	Indole-3-acetic acid
IL-1β	Interleukin-1β
IL-6	Interleukin-6
IxS	Indoxyl sulfate
MATE	Multidrug and toxin extrusion protein
mRNA	Messenger ribonucleic acid
MRP	Multidrug resistance-associated proteins
MW	Molecular weight
NF-κB	Nuclear factor kappa B
NO	Nitric oxide
Nx	Nephrectomy
OAT1	Organic anion transporter 1
OATP	Organic anion-transporting polypeptides
OCT	Organic cation transporter
PBUT	Protein-bound uremic toxin
pCG	*p*-cresyl glucuronide
pCS	*p*-cresyl sulfate
P-gp	P-glycoprotein
PTC	Proximal tubular cells
RAAS	Renin–angiotensin–aldosterone system
ROS	Reactive oxygen species
RSST	Remote sensing and signaling theory
SHBG	Sex hormone-binding globulin
SULT	Sulfotransferase
TMAO	Trimethylamine N-oxide
TNF-α	tumor necrosis factor-α
UA	Uric acid
UDP	Uridine diphosphate
UGT	UDP-glucuronosyltransferase
URAT	Urate transporter
UT	Uremic toxin
VC	Vascular calcification

## References

[1] Lohia S., Vlahou A., Zoidakis J. (2022). Microbiome in Chronic Kidney Disease (CKD): An Omics Perspective. Toxins.

[2] Bikbov B., Purcell C.A., Levey A.S., Smith M., Abdoli A., Abebe M., Adebayo O.M., Afarideh M., Agarwal S.K., Agudelo-Botero M. (2020). Global, regional, and national burden of chronic kidney disease, 1990–2017: A systematic analysis for the Global Burden of Disease Study 2017. Lancet.

[3] Pina-Beltran B., Dimitrov D., McKay N., Giot M., Zdráhal Z., Potěšil D., Pustka V., Peinado-Izaguerri J., Saez-Rodriguez J., Poitevin S. (2025). Unveiling the role of sex in the metabolism of indoxyl sulfate and apixaban. Sci. Rep..

[4] Andrassy K.M. (2013). Comments on ‘KDIGO 2012 Clinical Practice Guideline for the Evaluation and Management of Chronic Kidney Disease’. Kidney Int..

[5] National Kidney Foundation (2002). K/DOQI clinical practice guidelines for chronic kidney disease: Evaluation, classification, and stratification. Am. J. Kidney Dis..

[6] Webster A.C., Nagler E.V., Morton R.L., Masson P. (2017). Chronic Kidney Disease. Lancet.

[7] Shlipak M.G., Tummalapalli S.L., Boulware L.E., Grams M.E., Ix J.H., Jha V., Kengne A.P., Madero M., Mihaylova B., Tangri N. (2021). The case for early identification and intervention of chronic kidney disease: Conclusions from a Kidney Disease: Improving Global Outcomes (KDIGO) Controversies Conference. Kidney Int..

[8] Evenepoel P., Meijers B.K.I., Bammens B.R.M., Verbeke K. (2009). Uremic toxins originating from colonic microbial metabolism. Kidney Int..

[9] Vanholder R., Argilés A., Baurmeister U., Brunet P., Clark W., Cohen G., De Deyn P.P., Deppisch R., Descamps-Latscha B., Henle T. (2001). Uremic toxicity: Present state of the art. Int. J. Artif. Organs.

[10] Vanholder R., Pletinck A., Schepers E., Glorieux G. (2018). Biochemical and Clinical Impact of Organic Uremic Retention Solutes: A Comprehensive Update. Toxins.

[11] Gryp T., De Paepe K., Vanholder R., Kerckhof F.M., Van Biesen W., Van de Wiele T., Verbeke F., Speeckaert M., Joossens M., Couttenye M.M. (2020). Gut microbiota generation of protein-bound uremic toxins and related metabolites is not altered at different stages of chronic kidney disease. Kidney Int..

[12] Poesen R., Evenepoel P., de Loor H., Kuypers D., Augustijns P., Meijers B. (2016). Metabolism, Protein Binding, and Renal Clearance of Microbiota-Derived p-Cresol in Patients with CKD. Clin. J. Am. Soc. Nephrol..

[13] Sarnak M.J., Levey A.S., Schoolwerth A.C., Coresh J., Culleton B., Hamm L.L., McCullough P.A., Kasiske B.L., Kelepouris E., Klag M.J. (2003). Kidney disease as a risk factor for development of cardiovascular disease: A statement from the American Heart Association Councils on Kidney in Cardiovascular Disease, High Blood Pressure Research, Clinical Cardiology, and Epidemiology and Prevention. Hypertension.

[14] Glorieux G., Burtey S., Evenepoel P., Jankowski J., Koppe L., Masereeuw R., Vanholder R. (2026). A guide to uraemic toxicity. Nat. Rev. Nephrol..

[15] Hung S.C., Kuo K.L., Wu C.C., Tarng D.C. (2017). Indoxyl Sulfate: A Novel Cardiovascular Risk Factor in Chronic Kidney Disease. J. Am. Heart Assoc..

[16] Lekawanvijit S. (2018). Cardiotoxicity of Uremic Toxins: A Driver of Cardiorenal Syndrome. Toxins.

[17] Zhao Y., Yang K., Nguyen C.M., Wu H., Liu H., Velez L.M., Kim J.K., Seldin M., Lau W.L. (2025). Sex-specific cardiac dysfunction in mice with chronic kidney disease. Nephrol. Dial. Transplant..

[18] Lu Y.C., Wu C.C., Tsai I.T., Hung W.C., Lee T.L., Hsuan C.F., Yu T.H., Wei C.T., Chung F.M., Lee Y.J. (2021). Associations among total p-cresylsulfate, indoxyl sulfate and hippuric acid levels with hemodialysis quality indicators in maintenance hemodialysis patients. Clin. Chim. Acta.

[19] Faucher Q., van der Made T.K., De Lange E., Masereeuw R. (2023). Blood-brain barrier perturbations by uremic toxins: Key contributors in chronic kidney disease-induced neurological disorders?. Eur. J. Pharm. Sci..

[20] Schoots A.C., De Vries P.M., Thiemann R., Hazejager W.A., Visser S.L., Oe P.L. (1989). Biochemical and neurophysiological parameters in hemodialyzed patients with chronic renal failure. Clin. Chim. Acta.

[21] Hamed S.A. (2019). Neurologic conditions and disorders of uremic syndrome of chronic kidney disease: Presentations, causes, and treatment strategies. Expert Rev. Clin. Pharmacol..

[22] Barreto F.C., Barreto D.V., Liabeuf S., Drüeke T.B., Massy Z.A. (2009). Effects of uremic toxins on vascular and bone remodeling. Semin. Dial..

[23] Wu I.W., Hsu K.H., Lee C.C., Sun C.Y., Hsu H.J., Tsai C.J., Tzen C.Y., Wang Y.C., Lin C.Y., Wu M.S. (2011). p-Cresyl sulphate and indoxyl sulphate predict progression of chronic kidney disease. Nephrol. Dial. Transplant..

[24] Massy Z.A., Chesnaye N.C., Larabi I.A., Dekker F.W., Evans M., Caskey F.J., Torino C., Porto G., Szymczak M., Drechsler C. (2022). The relationship between uremic toxins and symptoms in older men and women with advanced chronic kidney disease. Clin. Kidney J..

[25] Ito S., Yoshida M. (2014). Protein-bound uremic toxins: New culprits of cardiovascular events in chronic kidney disease patients. Toxins.

[26] Magnani S., Atti M. (2021). Uremic Toxins and Blood Purification: A Review of Current Evidence and Future Perspectives. Toxins.

[27] Vanholder R.C., Eloot S., Glorieux G.L.R.L. (2016). Future Avenues to Decrease Uremic Toxin Concentration. Am. J. Kidney Dis..

[28] Ahmed S., Sparidans R.W., Vernooij R.W.M., Mihaila S.M., Broekhuizen R., Goldschmeding R., Nguyen T.Q., Masereeuw R., Gerritsen K.G.F. (2025). Protein-bound uremic toxin clearance as biomarker of kidney tubular function in diabetic kidney disease. Sci. Rep..

[29] Ahmed S., Chen S.X.L., Lucas J.P.F.E., Knoppert S.N., Harwood R., Faria J., Besseling P.J., Sparidans R.W., Broekhuizen R., Westphal K.G.C. (2025). Indoxyl sulfate as a potential tubular function marker across kidney disease models. Am. J. Physiol. Ren. Physiol..

[30] Wulczyn K.E., Shafi T., Anderson A., Rincon-Choles H., Clish C.B., Denburg M., Feldman H.I., He J., Hsu C.Y., Kelly T. (2024). Metabolites Associated With Uremic Symptoms in Patients With CKD: Findings From the Chronic Renal Insufficiency Cohort (CRIC) Study. Am. J. Kidney Dis..

[31] Short K.M., Smyth I.M. (2015). A morphological investigation of sexual and lateral dimorphism in the developing metanephric kidney. Sci. Rep..

[32] Steiger S., Li L., Bruchfeld A., Stevens K.I., Moran S.M., Floege J., Caravaca-Fontán F., Mirioglu S., Teng O.Y.K., Frangou E. (2025). Sex dimorphism in kidney health and disease: Mechanistic insights and clinical implication. Kidney Int..

[33] Murphy D., McCulloch C.E., Lin F., Banerjee T., Bragg-Gresham J.L., Eberhardt M.S., Morgenstern H., Pavkov M.E., Saran R., Powe N.R. (2016). Trends in Prevalence of Chronic Kidney Disease in the United States. Ann. Intern. Med..

[34] Kitai Y., Toriu N., Yoshikawa T., Sahara Y., Kinjo S., Shimizu Y., Sato Y., Oguchi A., Yamada R., Kondo M. (2025). Female sex hormones inversely regulate acute kidney disease susceptibility throughout life. Kidney Int..

[35] Carrero J.J., Hecking M., Chesnaye N.C., Jager K.J. (2018). Sex and gender disparities in the epidemiology and outcomes of chronic kidney disease. Nat. Rev. Nephrol..

[36] Valdivielso J.M., Jacobs-Cachá C., Soler M.J. (2019). Sex hormones and their influence on chronic kidney disease. Curr. Opin. Nephrol. Hypertens..

[37] Balafa O., Fernandez-Fernandez B., Ortiz A., Dounousi E., Ekart R., Ferro C.J., Mark P.B., Valdivielso J.M., Del Vecchio L., Mallamaci F. (2024). Sex disparities in mortality and cardiovascular outcomes in chronic kidney disease. Clin. Kidney J..

[38] Ricardo A.C., Yang W., Sha D., Appel L.J., Chen J., Krousel-Wood M., Manoharan A., Steigerwalt S., Wright J., Rahman M. (2019). Sex-Related Disparities in CKD Progression. J. Am. Soc. Nephrol..

[39] Joseph S., Nicolson T.J., Hammons G., Word B., Green-Knox B., Lyn-Cook B. (2015). Expression of drug transporters in human kidney: Impact of sex, age, and ethnicity. Biol. Sex Differ..

[40] Zou W., Shi B., Zeng T., Zhang Y., Huang B., Ouyang B., Cai Z., Liu M. (2021). Drug Transporters in the Kidney: Perspectives on Species Differences, Disease Status, and Molecular Docking. Front. Pharmacol..

[41] Veser C., Carlier A., Dubois V., Mihăilă S.M., Swapnasrita S. (2024). Embracing sex-specific differences in engineered kidney models for enhanced biological understanding of kidney function. Biol. Sex Differ..

[42] Poulsen C.G., Kjaergaard K.D., Peters C.D., Jespersen B., Jensen J.D. (2017). Quality of life development during initial hemodialysis therapy and association with loss of residual renal function. Hemodial. Int..

[43] Kotanko P., Thijssen S., Kitzler T., Wystrychowski G., Sarkar S.R., Zhu F., Gotch F., Levin N.W. (2007). Size matters: Body composition and outcomes in maintenance hemodialysis patients. Blood Purif..

[44] Ha S., Son M., Kim J., Kim D., Kim M.J., Yoo J., Kim B.M., Kim D., Chung H.Y., Chung K.W. (2025). Gender Differences in Adenine Diet-Induced Kidney Toxicity: The Impact of 17β-Estradiol on Renal Inflammation and Fibrosis. Int. J. Mol. Sci..

[45] Cozzolino M., Magagnoli L., Ciceri P. (2025). From Physicochemical Classification to Multidimensional Insights: A Comprehensive Review of Uremic Toxin Research. Toxins.

[46] Evenepoel P., Poesen R., Meijers B. (2017). The gut-kidney axis. Pediatr. Nephrol..

[47] Yang T., Richards E.M., Pepine C.J., Raizada M.K. (2018). The gut microbiota and the brain-gut-kidney axis in hypertension and chronic kidney disease. Nat. Rev. Nephrol..

[48] Bush K.T., Wu W., Lun C., Nigam S.K. (2017). The drug transporter OAT3 (SLC22A8) and endogenous metabolite communication via the gut-liver-kidney axis. J. Biol. Chem..

[49] Lowenstein J., Nigam S.K. (2021). Uremic Toxins in Organ Crosstalk. Front. Med..

[50] Calcaterra V., Rossi V., Massini G., Regalbuto C., Hruby C., Panelli S., Bandi C., Zuccotti G. (2022). Precocious puberty and microbiota: The role of the sex hormone-gut microbiome axis. Front. Endocrinol..

[51] Clotet-Freixas S., Zaslaver O., Kotlyar M., Pastrello C., Quaile A.T., McEvoy C.M., Saha A.D., Farkona S., Boshart A., Zorcic K. (2024). Sex differences in kidney metabolism may reflect sex-dependent outcomes in human diabetic kidney disease. Sci. Transl. Med..

[52] Li G., Xu Z., Yang H., Zhang D., Liu B., Song Y., Li Q., Zhang Y., Zhou H., Wang Y. (2026). Sex-specific mechanisms in the pathogenesis and progression of chronic kidney disease. Autoimmun. Rev..

[53] Ljubojevic M., Herak-Kramberger C.M., Hagos Y., Bahn A., Endou H., Burckhardt G., Sabolic I. (2004). Rat renal cortical OAT1 and OAT3 exhibit gender differences determined by both androgen stimulation and estrogen inhibition. Am. J. Physiol. Ren. Physiol..

[54] Looijer-van Langen M., Hotte N., Dieleman L.A., Albert E., Mulder C., Madsen K.L. (2011). Estrogen receptor-β signaling modulates epithelial barrier function. Am. J. Physiol. Gastrointest. Liver Physiol..

[55] Menon R., Watson S.E., Thomas L.N., Allred C.D., Dabney A., Azcarate-Peril M.A., Sturino J.M. (2013). Diet complexity and estrogen receptor β status affect the composition of the murine intestinal microbiota. Appl. Environ. Microbiol..

[56] Lichtenecker D.C.K., Argeri R., Castro C.H.d.M., Dias-da-Silva M.R., Gomes G.N. (2021). Cross-sex testosterone therapy modifies the renal morphology and function in female rats and might underlie increased systolic pressure. Clin. Exp. Pharmacol. Physiol..

[57] Oh E.S., Steele C.N., You Z., Nowak K.L., Jovanovich A.J. (2022). Sex hormones and the risk of cardiovascular disease and mortality in male and female patients with chronic kidney disease: A systematic review and meta-analysis. Physiol. Rep..

[58] Shin J.H., Park Y.H., Sim M., Kim S.A., Joung H., Shin D.M. (2019). Serum level of sex steroid hormone is associated with diversity and profiles of human gut microbiome. Res. Microbiol..

[59] Zeginiadou T., Kolias S., Kouretas D., Antonoglou O. (1997). Nonlinear binding of sex steroids to albumin and sex hormone binding globulin. Eur. J. Drug Metab. Pharmacokinet..

[60] van Eeghen S.A., Pyle L., Narongkiatikhun P., Choi Y.J., Obeid W., Parikh C.R., Vosters T.G., van Valkengoed I.G., Krebber M.M., Touw D.J. (2025). Unveiling mechanisms underlying kidney function changes during sex hormone therapy. J. Clin. Investig..

[61] Cobo G., Hecking M., Port F.K., Exner I., Lindholm B., Stenvinkel P., Carrero J.J. (2016). Sex and gender differences in chronic kidney disease: Progression to end-stage renal disease and haemodialysis. Clin. Sci..

[62] Hammes S.R., Levin E.R. (2019). Impact of estrogens in males and androgens in females. J. Clin. Investig..

[63] Almeida M., Laurent M.R., Dubois V., Claessens F., O’Brien C.A., Bouillon R., Vanderschueren D., Manolagas S.C. (2017). Estrogens and Androgens in Skeletal Physiology and Pathophysiology. Physiol. Rev..

[64] Laurent M., Antonio L., Sinnesael M., Dubois V., Gielen E., Classens F., Vanderschueren D. (2014). Androgens and estrogens in skeletal sexual dimorphism. Asian J. Androl..

[65] Soldin S.J., Soldin O.P. (2009). Steroid hormone analysis by tandem mass spectrometry. Clin. Chem..

[66] Sabolić I., Asif A.R., Budach W.E., Wanke C., Bahn A., Burckhardt G. (2007). Gender differences in kidney function. Pflug. Arch..

[67] Bennett N.C., Rajandram R., Ng K.L., Gobe G.C. (2014). Evaluation of steroid hormones and their receptors in development and progression of renal cell carcinoma. J. Kidney Cancer VHL.

[68] Conte C., Antonelli G., Melica M.E., Tarocchi M., Romagnani P., Peired A.J. (2023). Role of Sex Hormones in Prevalent Kidney Diseases. Int. J. Mol. Sci..

[69] Sharma P.K., Thakur M.K. (2004). Estrogen receptor alpha expression in mice kidney shows sex differences during aging. Biogerontology.

[70] Wilson C.M., McPhaul M.J. (1996). A and B forms of the androgen receptor are expressed in a variety of human tissues. Mol. Cell. Endocrinol..

[71] Antus B., Yao Y., Song E., Liu S., Lutz J., Heemann U. (2002). Opposite effects of testosterone and estrogens on chronic allograft nephropathy. Transpl. Int..

[72] Kang D.H., Yu E.S., Yoon K.I., Johnson R. (2004). The impact of gender on progression of renal disease: Potential role of estrogen-mediated vascular endothelial growth factor regulation and vascular protection. Am. J. Pathol..

[73] Quinkler M., Bumke-Vogt C., Meyer B., Bähr V., Oelkers W., Diederich S. (2003). The human kidney is a progesterone-metabolizing and androgen-producing organ. J. Clin. Endocrinol. Metab..

[74] Lu H., Lei X., Klaassen C. (2006). Gender differences in renal nuclear receptors and aryl hydrocarbon receptor in 5/6 nephrectomized rats. Kidney Int..

[75] Laouari D., Vergnaud P., Hirose T., Zaidan M., Rabant M., Nguyen C., Burtin M., Legendre C., Codogno P., Friedlander G. (2022). The sexual dimorphism of kidney growth in mice and humans. Kidney Int..

[76] McDonough A.A., Harris A.N., Xiong L.I., Layton A.T. (2024). Sex differences in renal transporters: Assessment and functional consequences. Nat. Rev. Nephrol..

[77] Conte C., Angelotti M.L., Mazzinghi B., Melica M.E., Antonelli G., Carangelo G., Landini S., Raglianti V., Ravaglia F., Cirillo L. (2025). Estrogen-regulated renal progenitors determine pregnancy adaptation and preeclampsia. Science.

[78] Veiras L.C., Girardi A.C.C., Curry J., Pei L., Ralph D.L., Tran A., Castelo-Branco R.C., Pastor-Soler N., Arranz C.T., Yu A.S.L. (2017). Sexual Dimorphic Pattern of Renal Transporters and Electrolyte Homeostasis. J. Am. Soc. Nephrol..

[79] Layton A.T., Sullivan J.C. (2019). Recent advances in sex differences in kidney function. Am. J. Physiol. Ren. Physiol..

[80] Li Q., McDonough A.A., Layton H.E., Layton A.T. (2018). Functional implications of sexual dimorphism of transporter patterns along the rat proximal tubule: Modeling and analysis. Am. J. Physiol. Ren. Physiol..

[81] Baylis C. (2012). Sexual dimorphism: The aging kidney, involvement of nitric oxide deficiency, and angiotensin II overactivity. J. Gerontol. A Biol. Sci. Med. Sci..

[82] Melsom T., Norvik J.V., Enoksen I.T., Stefansson V., Mathisen U.D., Fuskevåg O.M., Jenssen T.G., Solbu M.D., Eriksen B.O. (2022). Sex Differences in Age-Related Loss of Kidney Function. J. Am. Soc. Nephrol..

[83] Saastad V., Rinde L.B., Enoksen I.T., Porrini E., Eriksen B.O., Melsom T. (2025). Sex Differences in Kidney Function Decline in the Healthy General Population. Nephron.

[84] Hilliard L.M., Sampson A.K., Brown R.D., Denton K.M. (2013). The “his and hers” of the renin-angiotensin system. Curr. Hypertens. Rep..

[85] Ho L.T., Sprague S.M. (2013). Women and CKD-mineral and bone disorder. Adv. Chronic Kidney Dis..

[86] Curtis L.M. (2024). Sex and Gender Differences in AKI. Kidney360.

[87] Wei Q., Wang M.H., Dong Z. (2005). Differential gender differences in ischemic and nephrotoxic acute renal failure. Am. J. Nephrol..

[88] Dousdampanis P., Trigka K., Fourtounas C., Bargman J.M. (2014). Role of testosterone in the pathogenesis, progression, prognosis and comorbidity of men with chronic kidney disease. Ther. Apher. Dial..

[89] Nwia S.M., Leite A.P.O., Li X.C., Zhuo J.L. (2023). Sex differences in the renin-angiotensin-aldosterone system and its roles in hypertension, cardiovascular, and kidney diseases. Front. Cardiovasc. Med..

[90] Mauvais-Jarvis F., Lindsey S.H. (2024). Metabolic benefits afforded by estradiol and testosterone in both sexes: Clinical considerations. J. Clin. Investig..

[91] Yeo J.K., Koo H.S., Yu J., Park M.G. (2020). Effects of Testosterone Treatment on Quality of Life in Patients With Chronic Kidney Disease. Am. J. Mens. Health.

[92] Simpson E.R. (2002). Aromatization of androgens in women: Current concepts and findings. Fertil. Steril..

[93] van der Burgh A.C., Aribas E., Ikram M.A., Kavousi M., Neggers S.J.C.M.M., Hoorn E.J., Chaker L. (2023). Sex Differences in the Association Between Serum Testosterone and Kidney Function in the General Population. Kidney Int. Rep..

[94] van der Burgh A.C., Khan S.R., Neggers S., Hoorn E.J., Chaker L. (2022). The role of serum testosterone and dehydroepiandrosterone sulfate in kidney function and clinical outcomes in chronic kidney disease: A systematic review and meta-analysis. Endocr. Connect..

[95] Edey M.M. (2017). Male Sexual Dysfunction and Chronic Kidney Disease. Front. Med..

[96] Hajdu A., Rona G. (1971). The protective effect of estrogens against spontaneous pancratic islet and renal changes in aging male rats. Experientia.

[97] Silbiger S.R., Neugarten J. (1995). The impact of gender on the progression of chronic renal disease. Am. J. Kidney Dis..

[98] Ahmed S.B., Culleton B.F., Tonelli M., Klarenbach S.W., Macrae J.M., Zhang J., Hemmelgarn B.R. (2008). Oral estrogen therapy in postmenopausal women is associated with loss of kidney function. Kidney Int..

[99] Agarwal M., Selvan V., Freedman B.I., Liu Y., Wagenknecht L.E. (2005). The relationship between albuminuria and hormone therapy in postmenopausal women. Am. J. Kidney Dis..

[100] Kattah A.G., Suarez M.L.G., Milic N., Kantarci K., Zeydan B., Mosley T., Turner S.T., Ware E.B., Kardia S.L.R., Garovic V.D. (2018). Hormone therapy and urine protein excretion: A multiracial cohort study, systematic review, and meta-analysis. Menopause.

[101] Krupka E., Curtis S., Ferguson T., Whitlock R., Askin N., Millar A.C., Dahl M., Fung R., Ahmed S.B., Tangri N. (2022). The Effect of Gender-Affirming Hormone Therapy on Measures of Kidney Function: A Systematic Review and Meta-Analysis. Clin. J. Am. Soc. Nephrol..

[102] Zimmerman M.A., Ogola B.O., Wilkinson M.M., Visniauskas B., De Miguel C., Daniel J.M., Lindsey S.H. (2020). Medroxyprogesterone opposes estradiol-induced renal damage in midlife ovariectomized Long Evans rats. Menopause.

[103] Mineiro R., Santos C., Gonçalves I., Lemos M., Cavaco J.E.B., Quintela T. (2023). Regulation of ABC transporters by sex steroids may explain differences in drug resistance between sexes. J. Physiol. Biochem..

[104] Connelly P.J., Casey H., Montezano A.C., Touyz R.M., Delles C. (2022). Sex steroids receptors, hypertension, and vascular ageing. J. Hum. Hypertens..

[105] Wierman M.E. (2007). Sex steroid effects at target tissues: Mechanisms of action. Adv. Physiol. Educ..

[106] Neugarten J., Golestaneh L. (2019). Influence of Sex on the Progression of Chronic Kidney Disease. Mayo Clin. Proc..

[107] Piani F., Melena I., Tommerdahl K.L., Nokoff N., Nelson R.G., Pavkov M.E., van Raalte D.H., Cherney D.Z., Johnson R.J., Nadeau K.J. (2021). Sex-related differences in diabetic kidney disease: A review on the mechanisms and potential therapeutic implications. J. Diabetes Complicat..

[108] Mitchell T., De Miguel C., Gohar E.Y. (2020). Sex differences in redox homeostasis in renal disease. Redox Biol..

[109] Li L., Ju H., Jin H., Chen H., Sun M., Zhou Z. (2022). Low Testosterone Level and Risk of Adverse Clinical Events among Male Patients with Chronic Kidney Disease: A Systematic Review and Meta-Analysis of Cohort Studies. J. Healthc. Eng..

[110] Grossmann M., Hoermann R., Ng Tang Fui M., Zajac J.D., Ierino F.L., Roberts M.A. (2015). Sex steroids levels in chronic kidney disease and kidney transplant recipients: Associations with disease severity and prediction of mortality. Clin. Endocrinol..

[111] Shiraki N., Nakashima A., Doi S., Carrero J.J., Sugiya N., Ueno T., Stenvinkel P., Kohno N., Masaki T. (2014). Low serum testosterone is associated with atherosclerosis in postmenopausal women undergoing hemodialysis. Clin. Exp. Nephrol..

[112] Dhindsa S., Miller M.G., McWhirter C.L., Mager D.E., Ghanim H., Chaudhuri A., Dandona P. (2010). Testosterone concentrations in diabetic and nondiabetic obese men. Diabetes Care.

[113] Zitzmann M. (2024). Testosterone deficiency and chronic kidney disease. J. Clin. Transl. Endocrinol..

[114] Doumouchtsis K.K., Perrea D.N., Doumouchtsis S.K. (2009). The impact of sex hormone changes on bone mineral deficit in chronic renal failure. Endocr. Res..

[115] Christakou C.D., Diamanti-Kandarakis E. (2008). Role of androgen excess on metabolic aberrations and cardiovascular risk in women with polycystic ovary syndrome. Womens Health.

[116] Stamellou E., Sterzer V., Alam J., Roumeliotis S., Liakopoulos V., Dounousi E. (2024). Sex-Specific Differences in Kidney Function and Blood Pressure Regulation. Int. J. Mol. Sci..

[117] Yamada S., Nakano T. (2023). Role of Chronic Kidney Disease (CKD)-Mineral and Bone Disorder (MBD) in the Pathogenesis of Cardiovascular Disease in CKD. J. Atheroscler. Thromb..

[118] van den Beld A.W., de Jong F.H., Grobbee D.E., Pols H.A., Lamberts S.W. (2000). Measures of bioavailable serum testosterone and estradiol and their relationships with muscle strength, bone density, and body composition in elderly men. J. Clin. Endocrinol. Metab..

[119] Adeli A., Swahn E., Lind L., Soderberg S., Blomberg A., Engström G., Östgren C.J., Jernberg T., Bergström G., Settergren M. (2026). Sex differences in the prevalence and risk factors for aortic valve calcification in the general population. Heart.

[120] Shen Y., Yu C. (2024). The Bone-Vascular Axis: A Key Player in Chronic Kidney Disease Associated Vascular Calcification. Kidney Dis..

[121] Evenepoel P., Stenvinkel P., Shanahan C., Pacifici R. (2023). Inflammation and gut dysbiosis as drivers of CKD–MBD. Nat. Rev. Nephrol..

[122] Li J.Y., Chassaing B., Tyagi A.M., Vaccaro C., Luo T., Adams J., Darby T.M., Weitzmann M.N., Mulle J.G., Gewirtz A.T. (2016). Sex steroid deficiency-associated bone loss is microbiota dependent and prevented by probiotics. J. Clin. Investig..

[123] Zhao Y., Tran T., Fang C., Paganini-Hill A., Dulkanchainun M., Mai E., Eprem L., Cribbs D., Fisher M., Lau W.L. (2025). Gut dysbiosis and brain microhemorrhages in young vs. aged mice with chronic kidney disease. Sci. Rep..

[124] David K., Narinx N., De Loor J., Antonio L., Vanderschueren D., Claessens F., Decallonne B., Evenepoel P. (2023). #4184 Male Chronic Kidney Disease Is a State of Premature Testicular Aging. Nephrol. Dial. Transplant..

[125] Rosner M.H., Reis T., Husain-Syed F., Vanholder R., Hutchison C., Stenvinkel P., Blankestijn P.J., Cozzolino M., Juillard L., Kashani K. (2021). Classification of Uremic Toxins and Their Role in Kidney Failure. Clin. J. Am. Soc. Nephrol..

[126] Glorieux G., Nigam S.K., Vanholder R., Verbeke F. (2023). Role of the Microbiome in Gut-Heart-Kidney Cross Talk. Circ. Res..

[127] Vanholder R., De Smet R., Glorieux G., Argilés A., Baurmeister U., Brunet P., Clark W., Cohen G., De Deyn P.P., Deppisch R. (2003). Review on uremic toxins: Classification, concentration, and interindividual variability. Kidney Int..

[128] Spicher P., Brazier F., Laville S.M., Liabeuf S., Kamel S., Culot M., Bodeau S. (2025). Transporter-Mediated Interactions Between Uremic Toxins and Drugs: A Hidden Driver of Toxicity in Chronic Kidney Disease. Int. J. Mol. Sci..

[129] Leong S.C., Sirich T.L. (2016). Indoxyl Sulfate-Review of Toxicity and Therapeutic Strategies. Toxins.

[130] Vanholder R., Glorieux G., Argiles A., Burtey S., Cohen G., Duranton F., Koppe L., Massy Z.A., Ortiz A., Masereeuw R. (2025). Metabolomics to Identify Unclassified Uremic Toxins: A Comprehensive Literature Review. Kidney Med..

[131] Mishima E., Fukuda S., Mukawa C., Yuri A., Kanemitsu Y., Matsumoto Y., Akiyama Y., Fukuda N.N., Tsukamoto H., Asaji K. (2017). Evaluation of the impact of gut microbiota on uremic solute accumulation by a CE-TOFMS-based metabolomics approach. Kidney Int..

[132] Konturek P.C., Haziri D., Brzozowski T., Hess T., Heyman S., Kwiecien S., Konturek S.J., Koziel J. (2015). Emerging role of fecal microbiota therapy in the treatment of gastrointestinal and extra-gastrointestinal diseases. J. Physiol. Pharmacol..

[133] Viaene L., Thijs L., Jin Y., Liu Y., Gu Y., Meijers B., Claes K., Staessen J., Evenepoel P. (2014). Heritability and clinical determinants of serum indoxyl sulfate and p-cresyl sulfate, candidate biomarkers of the human microbiome enterotype. PLoS ONE.

[134] Lin C.J., Wu V., Wu P.C., Wu C.J. (2015). Meta-Analysis of the Associations of p-Cresyl Sulfate (PCS) and Indoxyl Sulfate (IS) with Cardiovascular Events and All-Cause Mortality in Patients with Chronic Renal Failure. PLoS ONE.

[135] Lin C.J., Chen H.H., Pan C.F., Chuang C.K., Wang T.J., Sun F.J., Wu C.J. (2011). p-Cresylsulfate and indoxyl sulfate level at different stages of chronic kidney disease. J. Clin. Lab. Anal..

[136] Arumugam M., Raes J., Pelletier E., Le Paslier D., Yamada T., Mende D.R., Fernandes G.R., Tap J., Bruls T., Batto J.M. (2011). Enterotypes of the human gut microbiome. Nature.

[137] Kim K., Anderson E.M., Thome T., Lu G., Salyers Z.R., Cort T.A., O’Malley K.A., Scali S.T., Ryan T.E. (2021). Skeletal myopathy in CKD: A comparison of adenine-induced nephropathy and 5/6 nephrectomy models in mice. Am. J. Physiol. Ren. Physiol..

[138] Hukriede N.A., Soranno D.E., Sander V., Perreau T., Starr M.C., Yuen P.S.T., Siskind L.J., Hutchens M.P., Davidson A.J., Burmeister D.M. (2022). Experimental models of acute kidney injury for translational research. Nat. Rev. Nephrol..

[139] Liang J., Liu Y. (2023). Animal Models of Kidney Disease: Challenges and Perspectives. Kidney360.

[140] Packialakshmi B., Stewart I.J., Burmeister D.M., Chung K.K., Zhou X. (2020). Large animal models for translational research in acute kidney injury. Ren. Fail..

[141] Nemours S., Castro L., Ribatallada-Soriano D., Semidey M.E., Aranda M., Ferrer M., Sanchez A., Morote J., Cantero-Recasens G., Meseguer A. (2022). Temporal and sex-dependent gene expression patterns in a renal ischemia–reperfusion injury and recovery pig model. Sci. Rep..

[142] Vanholder R., Nigam S.K., Burtey S., Glorieux G. (2022). What If Not All Metabolites from the Uremic Toxin Generating Pathways Are Toxic? A Hypothesis. Toxins.

[143] Lu Y., Meng L., Wang X., Zhang Y., Zhang C., Zhang M. (2025). The Non-Traditional Cardiovascular Culprits in Chronic Kidney Disease: Mineral Imbalance and Uremic Toxin Accumulation. Int. J. Mol. Sci..

[144] Rysz J., Franczyk B., Ławiński J., Olszewski R., Ciałkowska-Rysz A., Gluba-Brzózka A. (2021). The Impact of CKD on Uremic Toxins and Gut Microbiota. Toxins.

[145] Vanholder R., Glorieux G. (2018). Gut-Derived Metabolites and Chronic Kidney Disease: The Forest (F)or the Trees?. Clin. J. Am. Soc. Nephrol..

[146] Dou L., Bertrand E., Cerini C., Faure V., Sampol J., Vanholder R., Berland Y., Brunet P. (2004). The uremic solutes p-cresol and indoxyl sulfate inhibit endothelial proliferation and wound repair. Kidney Int..

[147] Liabeuf S., Glorieux G., Lenglet A., Diouf M., Schepers E., Desjardins L., Choukroun G., Vanholder R., Massy Z.A. (2013). Does p-cresylglucuronide have the same impact on mortality as other protein-bound uremic toxins?. PLoS ONE.

[148] Vanholder R., Schepers E., Pletinck A., Nagler E.V., Glorieux G. (2014). The uremic toxicity of indoxyl sulfate and p-cresyl sulfate: A systematic review. J. Am. Soc. Nephrol..

[149] Hwang I.K., Yoo K.Y., Li H., Park O.K., Lee C.H., Choi J.H., Jeong Y.G., Lee Y.L., Kim Y.M., Kwon Y.G. (2009). Indole-3-propionic acid attenuates neuronal damage and oxidative stress in the ischemic hippocampus. J. Neurosci. Res..

[150] Auld F., Maschauer E.L., Morrison I., Skene D.J., Riha R.L. (2017). Evidence for the efficacy of melatonin in the treatment of primary adult sleep disorders. Sleep Med. Rev..

[151] Chyan Y.J., Poeggeler B., Omar R.A., Chain D.G., Frangione B., Ghiso J., Pappolla M.A. (1999). Potent neuroprotective properties against the Alzheimer beta-amyloid by an endogenous melatonin-related indole structure, indole-3-propionic acid. J. Biol. Chem..

[152] Glorieux G., Gryp T., Perna A. (2020). Gut-Derived Metabolites and Their Role in Immune Dysfunction in Chronic Kidney Disease. Toxins.

[153] Shen Y., Fan N., Ma S.X., Cheng X., Yang X., Wang G. (2025). Gut Microbiota Dysbiosis: Pathogenesis, Diseases, Prevention, and Therapy. MedComm.

[154] Krukowski H., Valkenburg S., Vich Vila A., Maciel L.F., Vázquez-Castellanos J.F., Gryp T., Joossens M., Van Biesen W., Verbeke F., Derrien M. (2026). Host factors dictate gut microbiome alterations in chronic kidney disease more strongly than kidney function. Nat. Microbiol..

[155] Tsuji K., Uchida N., Nakanoh H., Fukushima K., Haraguchi S., Kitamura S., Wada J. (2025). The Gut–Kidney Axis in Chronic Kidney Diseases. Diagnostics.

[156] Anders H.J., Andersen K., Stecher B. (2013). The intestinal microbiota, a leaky gut, and abnormal immunity in kidney disease. Kidney Int..

[157] Huang Y., Xin W., Xiong J., Yao M., Zhang B., Zhao J. (2022). The Intestinal Microbiota and Metabolites in the Gut-Kidney-Heart Axis of Chronic Kidney Disease. Front. Pharmacol..

[158] Kim Y.S., Unno T., Kim B.Y., Park M.S. (2020). Sex Differences in Gut Microbiota. World J. Mens Health.

[159] Koren O., Goodrich J.K., Cullender T.C., Spor A., Laitinen K., Bäckhed H.K., Gonzalez A., Werner J.J., Angenent L.T., Knight R. (2012). Host remodeling of the gut microbiome and metabolic changes during pregnancy. Cell.

[160] Sinha T., Vich Vila A., Garmaeva S., Jankipersadsing S.A., Imhann F., Collij V., Bonder M.J., Jiang X., Gurry T., Alm E.J. (2019). Analysis of 1135 gut metagenomes identifies sex-specific resistome profiles. Gut Microbes.

[161] Cox-York K.A., Sheflin A.M., Foster M.T., Gentile C.L., Kahl A., Koch L.G., Britton S.L., Weir T.L. (2015). Ovariectomy results in differential shifts in gut microbiota in low versus high aerobic capacity rats. Physiol. Rep..

[162] Org E., Mehrabian M., Parks B.W., Shipkova P., Liu X., Drake T.A., Lusis A.J. (2016). Sex differences and hormonal effects on gut microbiota composition in mice. Gut Microbes.

[163] Lombardo M., Feraco A., Armani A., Camajani E., Gorini S., Strollo R., Padua E., Caprio M., Bellia A. (2024). Gender differences in body composition, dietary patterns, and physical activity: Insights from a cross-sectional study. Front. Nutr..

[164] Zhang P., Fang J., Li G., Zhang L., Lai X., Xu L., Liu L., Xiong Y., Li L., Zhang T. (2021). Sex Differences in Fecal Microbiota Correlation With Physiological and Biochemical Indices Associated With End-Stage Renal Disease Caused by Immunoglobulin a Nephropathy or Diabetes. Front. Microbiol..

[165] Lin T.-Y., Wu W.-K., Hung S.-C. (2025). High interindividual variability of indoxyl sulfate production identified by an oral tryptophan challenge test. npj Biofilms Microbiomes.

[166] James P., Stanford J., Dixit O.V.A., Nicdao M.A., McWhinney B., Sud K., Ryan M., Read S., Ahlenstiel G., Lambert K. (2025). Associations Between Uraemic Toxins and Gut Microbiota in Adults Initiating Peritoneal Dialysis. Toxins.

[167] Matz-Soja M., Berg T., Kietzmann T. (2026). Sex-related variations in liver homeostasis and disease: From zonation dynamics to clinical implications. J. Hepatol..

[168] Waxman D.J., Pampori N.A., Ram P.A., Agrawal A.K., Shapiro B.H. (1991). Interpulse interval in circulating growth hormone patterns regulates sexually dimorphic expression of hepatic cytochrome P450. Proc. Natl. Acad. Sci. USA.

[169] Yan Z., Zhong H.M., Maher N., Torres R., Leo G.C., Caldwell G.W., Huebert N. (2005). Bioactivation of 4-methylphenol (p-cresol) via cytochrome P450-mediated aromatic oxidation in human liver microsomes. Drug Metab. Dispos..

[170] Behal M., McCalla Z., Wang X. (2025). Sex differences in hepatic enzymes and transporters involved in pharmacokinetics. Liver Res..

[171] Yang L., Li Y., Hong H., Chang C.W., Guo L.W., Lyn-Cook B., Shi L., Ning B. (2012). Sex Differences in the Expression of Drug-Metabolizing and Transporter Genes in Human Liver. J. Drug Metab. Toxicol..

[172] Rong Y., Kiang T.K.L. (2020). Mechanisms of Metabolism Interaction Between p-Cresol and Mycophenolic Acid. Toxicol. Sci..

[173] Rong Y., Kiang T.K.L. (2020). Characterizations of Human UDP-Glucuronosyltransferase Enzymes in the Conjugation of p-Cresol. Toxicol. Sci..

[174] Kojima M., Degawa M. (2014). Sex differences in the constitutive gene expression of sulfotransferases and UDP-glucuronosyltransferases in the pig liver: Androgen-mediated regulation. Drug Metab. Pharmacokinet..

[175] Buckley D.B., Klaassen C.D. (2007). Tissue- and gender-specific mRNA expression of UDP-glucuronosyltransferases (UGTs) in mice. Drug Metab. Dispos..

[176] Hou H., Horikawa M., Narita Y., Jono H., Kakizoe Y., Izumi Y., Kuwabara T., Mukoyama M., Saito H. (2023). Suppression of Indoxyl Sulfate Accumulation Reduces Renal Fibrosis in Sulfotransferase 1a1-Deficient Mice. Int. J. Mol. Sci..

[177] Rong Y., Kiang T.K.L. (2021). Characterization of human sulfotransferases catalyzing the formation of p-cresol sulfate and identification of mefenamic acid as a potent metabolism inhibitor and potential therapeutic agent for detoxification. Toxicol. Appl. Pharmacol..

[178] Klaassen C.D., Liu L., Dunn R.T. (1998). Regulation of sulfotransferase mRNA expression in male and female rats of various ages. Chem.-Biol. Interact..

[179] D’Agostino G.D., Chaudhari S.N., Devlin A.S. (2024). Host-microbiome orchestration of the sulfated metabolome. Nat. Chem. Biol..

[180] Perna A.F., Glorieux G., Zacchia M., Trepiccione F., Capolongo G., Vigorito C., Anishchenko E., Ingrosso D. (2019). The role of the intestinal microbiota in uremic solute accumulation: A focus on sulfur compounds. J. Nephrol..

[181] Duranton F., Cohen G., De Smet R., Rodriguez M., Jankowski J., Vanholder R., Argiles A. (2012). Normal and pathologic concentrations of uremic toxins. J. Am. Soc. Nephrol..

[182] Cunha R.S.D., Azevedo C.A.B., Falconi C.A., Ruiz F.F., Liabeuf S., Carneiro-Ramos M.S., Stinghen A.E.M. (2022). The Interplay between Uremic Toxins and Albumin, Membrane Transporters and Drug Interaction. Toxins.

[183] Klammt S., Wojak H.-J., Mitzner A., Koball S., Rychly J., Reisinger E.C., Mitzner S. (2011). Albumin-binding capacity (ABiC) is reduced in patients with chronic kidney disease along with an accumulation of protein-bound uraemic toxins. Nephrol. Dial. Transplant..

[184] Lim Y.J., Sidor N.A., Tonial N.C., Che A., Urquhart B.L. (2021). Uremic Toxins in the Progression of Chronic Kidney Disease and Cardiovascular Disease: Mechanisms and Therapeutic Targets. Toxins.

[185] Weaving G., Batstone G.F., Jones R.G. (2016). Age and sex variation in serum albumin concentration: An observational study. Ann. Clin. Biochem..

[186] Adams S.V., Rivara M., Streja E., Cheung A.K., Arah O.A., Kalantar-Zadeh K., Mehrotra R. (2017). Sex Differences in Hospitalizations with Maintenance Hemodialysis. J. Am. Soc. Nephrol..

[187] Narinx N., David K., Walravens J., Vermeersch P., Claessens F., Fiers T., Lapauw B., Antonio L., Vanderschueren D. (2022). Role of sex hormone-binding globulin in the free hormone hypothesis and the relevance of free testosterone in androgen physiology. Cell. Mol. Life Sci..

[188] Oettl K., Stauber R.E. (2007). Physiological and pathological changes in the redox state of human serum albumin critically influence its binding properties. Br. J. Pharmacol..

[189] Neugarten J., Kasiske B., Silbiger S.R., Nyengaard J.R. (2002). Effects of sex on renal structure. Nephron.

[190] Toth-Manikowski S.M., Yang W., Appel L., Chen J., Deo R., Frydrych A., Krousel-Wood M., Rahman M., Rosas S.E., Sha D. (2021). Sex Differences in Cardiovascular Outcomes in CKD: Findings From the CRIC Study. Am. J. Kidney Dis..

[191] Kattah A.G., Garovic V.D. (2020). Understanding sex differences in progression and prognosis of chronic kidney disease. Ann. Transl. Med..

[192] Mutsaers H.A., Wilmer M.J., Reijnders D., Jansen J., van den Broek P.H., Forkink M., Schepers E., Glorieux G., Vanholder R., van den Heuvel L.P. (2013). Uremic toxins inhibit renal metabolic capacity through interference with glucuronidation and mitochondrial respiration. Biochim. Biophys. Acta.

[193] Mutsaers H.A., van den Heuvel L.P., Ringens L.H., Dankers A.C., Russel F.G., Wetzels J.F., Hoenderop J.G., Masereeuw R. (2011). Uremic toxins inhibit transport by breast cancer resistance protein and multidrug resistance protein 4 at clinically relevant concentrations. PLoS ONE.

[194] Buckley D.B., Klaassen C.D. (2009). Mechanism of gender-divergent UDP-glucuronosyltransferase mRNA expression in mouse liver and kidney. Drug Metab. Dispos..

[195] Liang J., Qiu Y., Fu T., Li J., Yang J., Tong Y. (2025). The Gut-Kidney Axis in Uric Acid Nephropathy: Microbiota, Metabolic Crosstalk, and Translational Prospects. J. Multidiscip. Healthc..

[196] Grant C.J., Harrison L.E., Hoad C.L., Marciani L., Gowland P.A., McIntyre C.W. (2017). Patients with chronic kidney disease have abnormal upper gastro-intestinal tract digestive function: A study of uremic enteropathy. J. Gastroenterol. Hepatol..

[197] Liabeuf S., Pepin M., Franssen C.F.M., Viggiano D., Carriazo S., Gansevoort R.T., Gesualdo L., Hafez G., Malyszko J., Mayer C. (2021). Chronic kidney disease and neurological disorders: Are uraemic toxins the missing piece of the puzzle?. Nephrol. Dial. Transplant..

[198] Adamczak M., Surma S. (2021). Metabolic Acidosis in Patients with CKD: Epidemiology, Pathogenesis, and Treatment. Kidney Dis..

[199] Owada S., Goto S., Bannai K., Hayashi H., Nishijima F., Niwa T. (2008). Indoxyl sulfate reduces superoxide scavenging activity in the kidneys of normal and uremic rats. Am. J. Nephrol..

[200] Motojima M., Hosokawa A., Yamato H., Muraki T., Yoshioka T. (2003). Uremic toxins of organic anions up-regulate PAI-1 expression by induction of NF-kappaB and free radical in proximal tubular cells. Kidney Int..

[201] Shimizu H., Bolati D., Adijiang A., Muteliefu G., Enomoto A., Nishijima F., Dateki M., Niwa T. (2011). NF-κB plays an important role in indoxyl sulfate-induced cellular senescence, fibrotic gene expression, and inhibition of proliferation in proximal tubular cells. Am. J. Physiol. Cell Physiol..

[202] Shimizu H., Bolati D., Higashiyama Y., Nishijima F., Shimizu K., Niwa T. (2012). Indoxyl sulfate upregulates renal expression of MCP-1 via production of ROS and activation of NF-κB, p53, ERK, and JNK in proximal tubular cells. Life Sci..

[203] Gryp T., Vanholder R., Vaneechoutte M., Glorieux G. (2017). p-Cresyl Sulfate. Toxins.

[204] Han H., Zhu J., Zhu Z., Ni J., Du R., Dai Y., Chen Y., Wu Z., Lu L., Zhang R. (2015). p-Cresyl sulfate aggravates cardiac dysfunction associated with chronic kidney disease by enhancing apoptosis of cardiomyocytes. J. Am. Heart Assoc..

[205] Mutsaers H.A., Caetano-Pinto P., Seegers A.E., Dankers A.C., van den Broek P.H., Wetzels J.F., van den Brand J.A., van den Heuvel L.P., Hoenderop J.G., Wilmer M.J. (2015). Proximal tubular efflux transporters involved in renal excretion of p-cresyl sulfate and p-cresyl glucuronide: Implications for chronic kidney disease pathophysiology. Toxicol. Vitr..

[206] Yao H., Zhao H., Du Y., Zhang Y., Li Y., Zhu H. (2024). Sex-related differences in SIRT3-mediated mitochondrial dynamics in renal ischemia/reperfusion injury. Transl. Res..

[207] Ricardo S.D., Zhang T., McArdle Z., Moore B.K., di Muzio A., Denton K.M., Widdop R.E. (2026). Sex-dependent differences in the progression of renal injury and fibrosis following ischemic acute kidney injury. Clin. Sci..

[208] Hyndman D., Liu S., Miner J.N. (2016). Urate Handling in the Human Body. Curr. Rheumatol. Rep..

[209] Falconi C.A., Junho C., Fogaça-Ruiz F., Vernier I.C.S., da Cunha R.S., Stinghen A.E.M., Carneiro-Ramos M.S. (2021). Uremic Toxins: An Alarming Danger Concerning the Cardiovascular System. Front. Physiol..

[210] Lekawanvijit S., Adrahtas A., Kelly D.J., Kompa A.R., Wang B.H., Krum H. (2010). Does indoxyl sulfate, a uraemic toxin, have direct effects on cardiac fibroblasts and myocytes?. Eur. Heart J..

[211] Lekawanvijit S., Kompa A.R., Manabe M., Wang B.H., Langham R.G., Nishijima F., Kelly D.J., Krum H. (2012). Chronic kidney disease-induced cardiac fibrosis is ameliorated by reducing circulating levels of a non-dialysable uremic toxin, indoxyl sulfate. PLoS ONE.

[212] Siroen M.P., Teerlink T., Nijveldt R.J., Prins H.A., Richir M.C., van Leeuwen P.A. (2006). The clinical significance of asymmetric dimethylarginine. Annu. Rev. Nutr..

[213] Aron-Wisnewsky J., Clément K. (2016). The gut microbiome, diet, and links to cardiometabolic and chronic disorders. Nat. Rev. Nephrol..

[214] Chen M.L., Zhu X.H., Ran L., Lang H.D., Yi L., Mi M.T. (2017). Trimethylamine-N-Oxide Induces Vascular Inflammation by Activating the NLRP3 Inflammasome Through the SIRT3-SOD2-mtROS Signaling Pathway. J. Am. Heart Assoc..

[215] Chinnappa S., Tu Y.K., Yeh Y.C., Glorieux G., Vanholder R., Mooney A. (2018). Association between Protein-Bound Uremic Toxins and Asymptomatic Cardiac Dysfunction in Patients with Chronic Kidney Disease. Toxins.

[216] Filipska I., Winiarska A., Knysak M., Stompór T. (2021). Contribution of Gut Microbiota-Derived Uremic Toxins to the Cardiovascular System Mineralization. Toxins.

[217] Hobson S., Qureshi A.R., Ripswedan J., Wennberg L., de Loor H., Ebert T., Söderberg M., Evenepoel P., Stenvinkel P., Kublickiene K. (2023). Phenylacetylglutamine and trimethylamine N-oxide: Two uremic players, different actions. Eur. J. Clin. Investig..

[218] Opdebeeck B., Maudsley S., Azmi A., De Maré A., De Leger W., Meijers B., Verhulst A., Evenepoel P., D’Haese P.C., Neven E. (2019). Indoxyl Sulfate and p-Cresyl Sulfate Promote Vascular Calcification and Associate with Glucose Intolerance. J. Am. Soc. Nephrol..

[219] Xue B., Johnson A.K., Hay M. (2013). Sex differences in angiotensin II- and aldosterone-induced hypertension: The central protective effects of estrogen. Am. J. Physiol. Regul. Integr. Comp. Physiol..

[220] Reddy Y.S., Kiranmayi V.S., Bitla A.R., Krishna G.S., Rao P.V., Sivakumar V. (2015). Nitric oxide status in patients with chronic kidney disease. Indian J. Nephrol..

[221] Reckelhoff J.F., Hennington B.S., Moore A.G., Blanchard E.J., Cameron J. (1998). Gender differences in the renal nitric oxide (NO) system: Dissociation between expression of endothelial NO synthase and renal hemodynamic response to NO synthase inhibition. Am. J. Hypertens..

[222] Kapil V., Rathod K.S., Khambata R.S., Bahra M., Velmurugan S., Purba A., Watson D.S., Barnes M.R., Wade W.G., Ahluwalia A. (2018). Sex differences in the nitrate-nitrite-NO• pathway: Role of oral nitrate-reducing bacteria. Free Radic. Biol. Med..

[223] Woodward H.J., Zhu D., Hadoke P.W.F., MacRae V.E. (2021). Regulatory Role of Sex Hormones in Cardiovascular Calcification. Int. J. Mol. Sci..

[224] Hung K.-C., Yao W.-C., Liu Y.-L., Yang H.-J., Liao M.-T., Chong K., Peng C.-H., Lu K.-C. (2023). The Potential Influence of Uremic Toxins on the Homeostasis of Bones and Muscles in Chronic Kidney Disease. Biomedicines.

[225] Anaya J.M., Bollag W.B., Hamrick M.W., Isales C.M. (2020). The Role of Tryptophan Metabolites in Musculoskeletal Stem Cell Aging. Int. J. Mol. Sci..

[226] Yamada S., Giachelli C.M. (2017). Vascular calcification in CKD-MBD: Roles for phosphate, FGF23, and Klotho. Bone.

[227] Cheung W.W., Zheng R., Hao S., Wang Z., Gonzalez A., Zhou P., Hoffman H.M., Mak R.H. (2021). The role of IL-1 in adipose browning and muscle wasting in CKD-associated cachexia. Sci. Rep..

[228] Wang X.H., Mitch W.E., Price S.R. (2022). Pathophysiological mechanisms leading to muscle loss in chronic kidney disease. Nat. Rev. Nephrol..

[229] Enoki Y., Watanabe H., Arake R., Sugimoto R., Imafuku T., Tominaga Y., Ishima Y., Kotani S., Nakajima M., Tanaka M. (2016). Indoxyl sulfate potentiates skeletal muscle atrophy by inducing the oxidative stress-mediated expression of myostatin and atrogin-1. Sci. Rep..

[230] Sato E., Mori T., Mishima E., Suzuki A., Sugawara S., Kurasawa N., Saigusa D., Miura D., Morikawa-Ichinose T., Saito R. (2016). Metabolic alterations by indoxyl sulfate in skeletal muscle induce uremic sarcopenia in chronic kidney disease. Sci. Rep..

[231] Haizlip K.M., Harrison B.C., Leinwand L.A. (2015). Sex-based differences in skeletal muscle kinetics and fiber-type composition. Physiology.

[232] Bredella M.A. (2017). Sex Differences in Body Composition. Adv. Exp. Med. Biol..

[233] Tayama Y., Ogawa T., Konishi Y., Takahashi K., Fujii K., Shintani A., Hasegawa H. (2025). Sex differences in the association between muscle mass or strength and nutrition status in chronic hemodialysis patients. Ren. Replace. Ther..

[234] Stenvinkel P., Barany P., Chung S.H., Lindholm B., Heimbürger O. (2002). A comparative analysis of nutritional parameters as predictors of outcome in male and female ESRD patients. Nephrol. Dial. Transplant..

[235] Kumchev E.P., Tzvetkova S.B., Enchev E.D., Yaneva M.P., Dimitrova R.H., Botushanova A.D., Dimitrakov D.J. (2000). Influence of age, sex and body weight on renal osteodystrophy in predialysis patients with chronic renal failure. Folia Med..

[236] Cheung A.K., Rocco M.V., Yan G., Leypoldt J.K., Levin N.W., Greene T., Agodoa L., Bailey J., Beck G.J., Clark W. (2006). Serum beta-2 microglobulin levels predict mortality in dialysis patients: Results of the HEMO study. J. Am. Soc. Nephrol..

[237] Locatelli F., Gauly A., Czekalski S., Hannedouche T., Jacobson S.H., Loureiro A., Martin-Malo A., Papadimitriou M., Passlick-Deetjen J., Ronco C. (2008). The MPO Study: Just a European HEMO Study or something very different?. Blood Purif..

[238] Tonkovic U., Bogicevic M., Manzar A., Andrejic N., Sic A., Atanaskovic M., Gajić S., Bontić A., Ksiazek S.H., Mijušković A. (2025). Neurological Manifestations of Hemolytic Uremic Syndrome: A Comprehensive Review. Brain Sci..

[239] Koek W.N.H., Campos-Obando N., van der Eerden B.C.J., de Rijke Y.B., Ikram M.A., Uitterlinden A.G., van Leeuwen J., Zillikens M.C. (2021). Age-dependent sex differences in calcium and phosphate homeostasis. Endocr. Connect..

[240] Franco Á.O., Starosta R.T., Roriz-Cruz M. (2019). The specific impact of uremic toxins upon cognitive domains: A review. J. Bras. Nefrol..

[241] Makhammajanov Z., Gaipov A., Myngbay A., Bukasov R., Aljofan M., Kanbay M. (2023). Tubular toxicity of proteinuria and the progression of chronic kidney disease. Nephrol. Dial. Transplant..

[242] Masereeuw R., Verhaar M.C. (2020). Innovations in approaches to remove uraemic toxins. Nat. Rev. Nephrol..

[243] Breljak D., Brzica H., Sweet D.H., Anzai N., Sabolic I. (2013). Sex-dependent expression of Oat3 (Slc22a8) and Oat1 (Slc22a6) proteins in murine kidneys. Am. J. Physiol. Ren. Physiol..

[244] Lu H., Klaassen C. (2008). Gender differences in mRNA expression of ATP-binding cassette efflux and bile acid transporters in kidney, liver, and intestine of 5/6 nephrectomized rats. Drug Metab. Dispos..

[245] Jansen J., Fedecostante M., Wilmer M.J., Peters J.G., Kreuser U.M., van den Broek P.H., Mensink R.A., Boltje T.J., Stamatialis D., Wetzels J.F. (2016). Bioengineered kidney tubules efficiently excrete uremic toxins. Sci. Rep..

[246] Wu W., Bush K.T., Nigam S.K. (2017). Key Role for the Organic Anion Transporters, OAT1 and OAT3, in the in vivo Handling of Uremic Toxins and Solutes. Sci. Rep..

[247] Yanai H., Katsuyama H., Hakoshima M., Adachi H. (2023). Urate Transporter 1 Can Be a Therapeutic Target Molecule for Chronic Kidney Disease and Diabetic Kidney Disease: A Retrospective Longitudinal Study. Biomedicines.

[248] Masereeuw R., Mutsaers H.A.M., Toyohara T., Abe T., Jhawar S., Sweet D.H., Lowenstein J. (2014). The Kidney and Uremic Toxin Removal: Glomerulus or Tubule?. Semin. Nephrol..

[249] Dalla C., Pavlidi P., Sakelliadou D.G., Grammatikopoulou T., Kokras N. (2022). Sex Differences in Blood-Brain Barrier Transport of Psychotropic Drugs. Front. Behav. Neurosci..

[250] Urakami Y., Nakamura N., Takahashi K., Okuda M., Saito H., Hashimoto Y., Inui K. (1999). Gender differences in expression of organic cation transporter OCT2 in rat kidney. FEBS Lett..

[251] Slitt A., Cherrington N., Hartley D., Leazer T., Klaassen C. (2002). Tissue Distribution and Renal Developmental Changes in Rat Organic Cation Transporter mRNA levels. Drug Metab. Dispos..

[252] Alnouti Y., Petrick J.S., Klaassen C.D. (2006). Tissue Distribution and Ontogeny of Organic Cation Transporters in Mice. Drug Metab. Dispos..

[253] Maher J.M., Cheng X., Tanaka Y., Scheffer G.L., Klaassen C.D. (2006). Hormonal regulation of renal multidrug resistance-associated proteins 3 and 4 (Mrp3 and Mrp4) in mice. Biochem. Pharmacol..

[254] Tanaka Y., Slitt A.L., Leazer T.M., Maher J.M., Klaassen C.D. (2005). Tissue distribution and hormonal regulation of the breast cancer resistance protein (Bcrp/Abcg2) in rats and mice. Biochem. Biophys. Res. Commun..

[255] Imai Y., Ishikawa E., Asada S., Sugimoto Y. (2005). Estrogen-mediated post transcriptional down-regulation of breast cancer resistance protein/ABCG2. Cancer Res..

[256] Takiue Y., Hosoyamada M., Kimura M., Saito H. (2011). The effect of female hormones upon urate transport systems in the mouse kidney. Nucleosides Nucleot. Nucl. Acids.

[257] Rezzani R., Franco C., Hardeland R., Rodella L.F. (2020). Thymus-Pineal Gland Axis: Revisiting Its Role in Human Life and Ageing. Int. J. Mol. Sci..

[258] Xiong L., Liu J., Han S.Y., Koppitch K., Guo J.J., Rommelfanger M., Miao Z., Gao F., Hallgrimsdottir I.B., Pachter L. (2023). Direct androgen receptor control of sexually dimorphic gene expression in the mammalian kidney. Dev. Cell.

[259] Lauriola M., Zadora W., Farré R., Meijers B. (2024). Intestinal transport of organic food compounds and drugs: A scoping review on the alterations observed in chronic kidney disease. Clin. Nutr. ESPEN.

[260] Naud J., Michaud J., Boisvert C., Desbiens K., Leblond F.A., Mitchell A., Jones C., Bonnardeaux A., Pichette V. (2007). Down-regulation of intestinal drug transporters in chronic renal failure in rats. J. Pharmacol. Exp. Ther..

[261] Veau C., Leroy C., Banide H., Auchère D., Tardivel S., Farinotti R., Lacour B. (2001). Effect of chronic renal failure on the expression and function of rat intestinal P-glycoprotein in drug excretion. Nephrol. Dial. Transplant..

[262] Tsujimoto M., Hatozaki D., Shima D., Yokota H., Furukubo T., Izumi S., Yamakawa T., Minegaki T., Nishiguchi K. (2012). Influence of serum in hemodialysis patients on the expression of intestinal and hepatic transporters for the excretion of pravastatin. Ther. Apher. Dial..

[263] Tatosian D.A., Yee K.L., Zhang Z., Mostoller K., Paul E., Sutradhar S., Larson P., Chhibber A., Wen J., Wang Y.J. (2021). A Microdose Cocktail to Evaluate Drug Interactions in Patients with Renal Impairment. Clin. Pharmacol. Ther..

[264] Yano H., Tamura Y., Kobayashi K., Tanemoto M., Uchida S. (2014). Uric acid transporter ABCG2 is increased in the intestine of the 5/6 nephrectomy rat model of chronic kidney disease. Clin. Exp. Nephrol..

[265] Mai Y., Dou L., Yao Z., Madla C.M., Gavins F.K.H., Taherali F., Yin H., Orlu M., Murdan S., Basit A.W. (2021). Quantification of P-Glycoprotein in the Gastrointestinal Tract of Humans and Rodents: Methodology, Gut Region, Sex, and Species Matter. Mol. Pharm..

[266] Madla C.M., Qin Y., Gavins F.K.H., Liu J., Dou L., Orlu M., Murdan S., Mai Y., Basit A.W. (2022). Sex Differences in Intestinal P-Glycoprotein Expression in Wistar versus Sprague Dawley Rats. Pharmaceutics.

[267] Gutmann H., Hruz P., Zimmermann C., Beglinger C., Drewe J. (2005). Distribution of breast cancer resistance protein (BCRP/ABCG2) mRNA expression along the human GI tract. Biochem. Pharmacol..

[268] Mai Y., Gavins F.K.H., Dou L., Liu J., Taherali F., Alkahtani M.E., Murdan S., Basit A.W., Orlu M. (2021). A Non-Nutritive Feeding Intervention Alters the Expression of Efflux Transporters in the Gastrointestinal Tract. Pharmaceutics.

[269] Naud J., Michaud J., Beauchemin S., Hébert M.J., Roger M., Lefrancois S., Leblond F.A., Pichette V. (2011). Effects of chronic renal failure on kidney drug transporters and cytochrome P450 in rats. Drug Metab. Dispos..

[270] Santana Machado T., Poitevin S., Paul P., McKay N., Jourde-Chiche N., Legris T., Mouly-Bandini A., Dignat-George F., Brunet P., Masereeuw R. (2018). Indoxyl Sulfate Upregulates Liver P-Glycoprotein Expression and Activity through Aryl Hydrocarbon Receptor Signaling. J. Am. Soc. Nephrol..

[271] Rost D., Kopplow K., Gehrke S., Mueller S., Friess H., Ittrich C., Mayer D., Stiehl A. (2005). Gender-specific expression of liver organic anion transporters in rat. Eur. J. Clin. Investig..

[272] Suzuki T., Zhao Y.L., Nadai M., Naruhashi K., Shimizu A., Takagi K., Takagi K., Hasegawa T. (2006). Gender-related differences in expression and function of hepatic P-glycoprotein and multidrug resistance-associated protein (Mrp2) in rats. Life Sci..

[273] Swann J.R., Spitzer S.O., Diaz Heijtz R. (2020). Developmental Signatures of Microbiota-Derived Metabolites in the Mouse Brain. Metabolites.

[274] Mahringer A., Fricker G. (2016). ABC transporters at the blood-brain barrier. Expert Opin. Drug Metab. Toxicol..

[275] Ohtsuki S., Tomi M., Hata T., Nagai Y., Hori S., Mori S., Hosoya K., Terasaki T. (2005). Dominant expression of androgen receptors and their functional regulation of organic anion transporter 3 in rat brain capillary endothelial cells; comparison of gene expression between the blood-brain and -retinal barriers. J. Cell. Physiol..

[276] Curran C.S., Kopp J.B. (2022). Aryl Hydrocarbon Receptor Mechanisms Affecting Chronic Kidney Disease. Front. Pharmacol..

[277] Madella A.M., Van Bergenhenegouwen J., Garssen J., Masereeuw R., Overbeek S.A. (2022). Microbial-Derived Tryptophan Catabolites, Kidney Disease and Gut Inflammation. Toxins.

[278] Mo Y., Lu Z., Wang L., Ji C., Zou C., Liu X. (2020). The Aryl Hydrocarbon Receptor in Chronic Kidney Disease: Friend or Foe?. Front. Cell Dev. Biol..

[279] Shinde R., McGaha T.L. (2018). The Aryl Hydrocarbon Receptor: Connecting Immunity to the Microenvironment. Trends Immunol..

[280] Dou L., Sallée M., Cerini C., Poitevin S., Gondouin B., Jourde-Chiche N., Fallague K., Brunet P., Calaf R., Dussol B. (2015). The cardiovascular effect of the uremic solute indole-3 acetic acid. J. Am. Soc. Nephrol..

[281] Gondouin B., Cerini C., Dou L., Sallée M., Duval-Sabatier A., Pletinck A., Calaf R., Lacroix R., Jourde-Chiche N., Poitevin S. (2013). Indolic uremic solutes increase tissue factor production in endothelial cells by the aryl hydrocarbon receptor pathway. Kidney Int..

[282] Dou L., Poitevin S., Sallée M., Addi T., Gondouin B., McKay N., Denison M.S., Jourde-Chiche N., Duval-Sabatier A., Cerini C. (2018). Aryl hydrocarbon receptor is activated in patients and mice with chronic kidney disease. Kidney Int..

[283] Kolachalama V.B., Shashar M., Alousi F., Shivanna S., Rijal K., Belghasem M.E., Walker J., Matsuura S., Chang G.H., Gibson C.M. (2018). Uremic Solute-Aryl Hydrocarbon Receptor-Tissue Factor Axis Associates with Thrombosis after Vascular Injury in Humans. J. Am. Soc. Nephrol..

[284] Candellier A., Issa N., Grissi M., Brouette T., Avondo C., Gomila C., Blot G., Gubler B., Touati G., On Behalf Of The Stop-As Investigators (2023). Indoxyl-sulfate activation of the AhR- NF-κB pathway promotes interleukin-6 secretion and the subsequent osteogenic differentiation of human valvular interstitial cells from the aortic valve. J. Mol. Cell. Cardiol..

[285] Bobot M., Thomas L., Moyon A., Fernandez S., McKay N., Balasse L., Garrigue P., Brige P., Chopinet S., Poitevin S. (2020). Uremic Toxic Blood-Brain Barrier Disruption Mediated by AhR Activation Leads to Cognitive Impairment during Experimental Renal Dysfunction. J. Am. Soc. Nephrol..

[286] Bobot M., Guedj E., Resseguier N., Faraut J., Garrigue P., Nail V., Hache G., Gonzalez S., McKay N., Vial R. (2024). Increased Blood-Brain Barrier Permeability and Cognitive Impairment in Patients With ESKD. Kidney Int. Rep..

[287] Haque N., Tischkau S.A. (2022). Sexual Dimorphism in Adipose-Hypothalamic Crosstalk and the Contribution of Aryl Hydrocarbon Receptor to Regulate Energy Homeostasis. Int. J. Mol. Sci..

[288] Liabeuf S., Cheddani L., Massy Z.A. (2018). Uremic Toxins and Clinical Outcomes: The Impact of Kidney Transplantation. Toxins.

[289] Dai L., Massy Z.A., Stenvinkel P., Chesnaye N.C., Larabi I.A., Alvarez J.C., Caskey F.J., Torino C., Porto G., Szymczak M. (2022). The association between TMAO, CMPF, and clinical outcomes in advanced chronic kidney disease: Results from the European QUALity (EQUAL) Study. Am. J. Clin. Nutr..

[290] Barreto F.C., Barreto D.V., Liabeuf S., Meert N., Glorieux G., Temmar M., Choukroun G., Vanholder R., Massy Z.A. (2009). Serum indoxyl sulfate is associated with vascular disease and mortality in chronic kidney disease patients. Clin. J. Am. Soc. Nephrol..

[291] Liabeuf S., Barreto D.V., Barreto F.C., Meert N., Glorieux G., Schepers E., Temmar M., Choukroun G., Vanholder R., Massy Z.A. (2010). Free p-cresylsulphate is a predictor of mortality in patients at different stages of chronic kidney disease. Nephrol. Dial. Transplant..

[292] Meijers B.K., Claes K., Bammens B., de Loor H., Viaene L., Verbeke K., Kuypers D., Vanrenterghem Y., Evenepoel P. (2010). p-Cresol and cardiovascular risk in mild-to-moderate kidney disease. Clin. J. Am. Soc. Nephrol..

[293] Reyes M., Benet L.Z. (2011). Effects of uremic toxins on transport and metabolism of different biopharmaceutics drug disposition classification system xenobiotics. J. Pharm. Sci..

[294] Arakawa H., Kato Y. (2023). Emerging Roles of Uremic Toxins and Inflammatory Cytokines in the Alteration of Hepatic Drug Disposition in Patients with Kidney Dysfunction. Drug Metab. Dispos..

[295] André C., Choukroun G., Bennis Y., Kamel S., Lemaire-Hurtel A.S., Masmoudi K., Bodeau S., Liabeuf S. (2022). Potential interactions between uraemic toxins and drugs: An application in kidney transplant recipients treated with calcineurin inhibitors. Nephrol. Dial. Transplant..

[296] Xiong J., Wang M., Wang J., Yang K., Shi Y., Zhang J., Zhang B., Zhang L., Zhao J. (2020). Geriatric nutrition risk index is associated with renal progression, cardiovascular events and all-cause mortality in chronic kidney disease. J. Nephrol..

[297] Thompson L.E., Joy M.S. (2022). Endogenous markers of kidney function and renal drug clearance processes of filtration, secretion, and reabsorption. Curr. Opin. Toxicol..

[298] Risso M.A., Sallustio S., Sueiro V., Bertoni V., Gonzalez-Torres H., Musso C.G. (2019). The Importance of Tubular Function in Chronic Kidney Disease. Int. J. Nephrol. Renov. Dis..

[299] Rhee E.P., Guallar E., Hwang S., Kim N., Tonelli M., Moe S.M., Himmelfarb J., Thadhani R.I., Powe N.R., Shafi T. (2020). Prevalence and Persistence of Uremic Symptoms in Incident Dialysis Patients. Kidney360.

[300] Hu J.-R., Myint L., Levey A.S., Coresh J., Inker L.A., Grams M.E., Guallar E., Hansen K.D., Rhee E.P., Shafi T. (2022). A metabolomics approach identified toxins associated with uremic symptoms in advanced chronic kidney disease. Kidney Int..

[301] Lin W., Chen Y., Unadkat J.D., Zhang X., Wu D., Heimbach T. (2022). Applications, Challenges, and Outlook for PBPK Modeling and Simulation: A Regulatory, Industrial and Academic Perspective. Pharm. Res..

[302] Lahane G.P., Dhar A., Bhat A. (2025). Model Systems of Chronic Kidney Disease: A Detailed Overview and Recent Advances. J. Biochem. Mol. Toxicol..

[303] Malik P.R.V., Yeung C.H.T., Ismaeil S., Advani U., Djie S., Edginton A.N. (2020). A Physiological Approach to Pharmacokinetics in Chronic Kidney Disease. J. Clin. Pharmacol..

[304] Dubinsky S., Malik P., Hajducek D.M., Edginton A. (2022). Determining the Effects of Chronic Kidney Disease on Organic Anion Transporter1/3 Activity Through Physiologically Based Pharmacokinetic Modeling. Clin. Pharmacokinet..

[305] Hsueh C.H., Hsu V., Zhao P., Zhang L., Giacomini K.M., Huang S.M. (2018). PBPK Modeling of the Effect of Reduced Kidney Function on the Pharmacokinetics of Drugs Excreted Renally by Organic Anion Transporters. Clin. Pharmacol. Ther..

[306] Scotcher D., Jones C.R., Galetin A., Rostami-Hodjegan A. (2017). Delineating the Role of Various Factors in Renal Disposition of Digoxin through Application of Physiologically Based Kidney Model to Renal Impairment Populations. J. Pharmacol. Exp. Ther..

[307] Tan S.P.F., Willemin M.E., Snoeys J., Shen H., Rostami-Hodjegan A., Scotcher D., Galetin A. (2023). Development of 4-Pyridoxic Acid PBPK Model to Support Biomarker-Informed Evaluation of OAT1/3 Inhibition and Effect of Chronic Kidney Disease. Clin. Pharmacol. Ther..

[308] Chang S.Y., Huang W., Chapron A., Quiñones A.J.L., Wang J., Isoherranen N., Shen D.D., Kelly E.J., Himmelfarb J., Yeung C.K. (2023). Incorporating Uremic Solute-mediated Inhibition of OAT1/3 Improves PBPK Prediction of Tenofovir Renal and Systemic Disposition in Patients with Severe Kidney Disease. Pharm. Res..

[309] Pak Y.A., Posada M.M., Bacon J., Long A., Annes W., Witcher J., Mitchell M., Tirona R.G., Hall S.D., Hillgren K.M. (2023). Prediction of the Renal Organic Anion Transporter 1 (OAT1)- Mediated Drug Interactions for LY404039, the Active Metabolite of Pomaglumetad Methionil. Pharm. Res..

[310] Asano S., Galetin A., Tomita Y., Giacomini K.M., Chu X., Yang X., Nakamura T., Kusuhara H., Sugiyama Y. (2025). Predicting OCT2/MATEs-Mediated Drug Interactions in Healthy Volunteers and Patients with Chronic Kidney Disease: Insights from Extended Clearance Concept, Endogenous Biomarkers, and In Vitro Inhibition Studies (Perspectives from the International Transporter Consortium). Clin. Pharmacol. Ther..

[311] King J., Mihaila S.M., Ahmed S., Truckenmüller R., Giselbrecht S., Masereeuw R., Carlier A. (2021). The Influence of OAT1 Density and Functionality on Indoxyl Sulfate Transport in the Human Proximal Tubule: An Integrated Computational and In Vitro Study. Toxins.

[312] Hu R., McDonough A.A., Layton A.T. (2019). Functional implications of the sex differences in transporter abundance along the rat nephron: Modeling and analysis. Am. J. Physiol. Ren. Physiol..

[313] Hu R., McDonough A.A., Layton A.T. (2020). Sex differences in solute transport along the nephrons: Effects of Na(+) transport inhibition. Am. J. Physiol. Ren. Physiol..

[314] Hu R., McDonough A.A., Layton A.T. (2021). Sex differences in solute and water handling in the human kidney: Modeling and functional implications. iScience.

[315] Burrowes K.S., Ruppage M., Lowry A., Zhao D. (2023). Sex matters: The frequently overlooked importance of considering sex in computational models. Front. Physiol..

[316] Bhargava A., Arnold A.P., Bangasser D.A., Denton K.M., Gupta A., Hilliard Krause L.M., Mayer E.A., McCarthy M., Miller W.L., Raznahan A. (2021). Considering Sex as a Biological Variable in Basic and Clinical Studies: An Endocrine Society Scientific Statement. Endocr. Rev..

[317] Castro-Aldrete L., Einsiedler M., Cuní-López C., Vanhaelen Q., Silvestri A., Ferretti M.T., de Gennaro M.E., Putignano G., Guix M., Marino N. (2026). Modelling sex differences of neurological disorders in vitro. Nat. Rev. Bioeng..

[318] (2026). Sex as a biological variable in biomedical engineering. Nat. Rev. Bioeng..

[319] Ilatovskaya D.V., Ogola B., Faulkner J.L., Mamenko M., Taylor E.B., Dent E., Ryan M.J., Sullivan J.C. (2025). Guidelines for sex-specific considerations to improve rigor in renal research and how we got there. Am. J. Physiol. Ren. Physiol..

[320] Soranno D.E., Awdishu L., Bagshaw S.M., Basile D., Bell S., Bihorac A., Bonventre J., Brendolan A., Claure-Del Granado R., Collister D. (2025). The role of sex and gender in acute kidney injury-consensus statements from the 33rd Acute Disease Quality Initiative. Kidney Int..

[321] Caetano-Pinto P., Stahl S.H. (2023). Renal Organic Anion Transporters 1 and 3 In Vitro: Gone but Not Forgotten. Int. J. Mol. Sci..

[322] Pou Casellas C., Jansen K., Rookmaaker M.B., Clevers H., Verhaar M.C., Masereeuw R. (2022). Regulation of solute carriers oct2 and OAT1/3 in the kidney: A phylogenetic, ontogenetic, and cell dynamic perspective. Physiol. Rev..

[323] Kim Y.K., Nam S.A., Yang C.W. (2018). Applications of kidney organoids derived from human pluripotent stem cells. Korean J. Intern. Med..

[324] Nakanoh H., Tsuji K., Fukushima K., Uchida N., Haraguchi S., Kitamura S., Wada J. (2025). Kidney Organoids: Current Advances and Applications. Life.

[325] Rizki-Safitri A., Traitteur T., Morizane R. (2021). Bioengineered Kidney Models: Methods and Functional Assessments. Function.

[326] Ashammakhi N., Wesseling-Perry K., Hasan A., Elkhammas E., Zhang Y.S. (2018). Kidney-on-a-chip: Untapped opportunities. Kidney Int..

[327] Zommiti M., Connil N., Tahrioui A., Groboillot A., Barbey C., Konto-Ghiorghi Y., Lesouhaitier O., Chevalier S., Feuilloley M.G.J. (2022). Organs-on-Chips Platforms Are Everywhere: A Zoom on Biomedical Investigation. Bioengineering.

